# Chronic inflammatory arthritis drives systemic changes in circadian energy metabolism

**DOI:** 10.1073/pnas.2112781119

**Published:** 2022-04-28

**Authors:** Polly Downton, Fabio Sanna, Robert Maidstone, Toryn M. Poolman, Edward A. Hayter, Suzanna H. Dickson, Nick A. Ciccone, James O. Early, Antony Adamson, David G. Spiller, Devin A. Simpkins, Matthew Baxter, Roman Fischer, Magnus Rattray, Andrew S. I. Loudon, Julie E. Gibbs, David A. Bechtold, David W. Ray

**Affiliations:** ^a^Centre for Biological Timing, Faculty of Biology, Medicine and Health, University of Manchester, Manchester, M13 9PT, United Kingdom;; ^b^National Institute for Health Research Oxford Biomedical Research Centre, John Radcliffe Hospital, Oxford, OX3 9DU, United Kingdom;; ^c^Oxford Centre for Diabetes, Endocrinology and Metabolism, University of Oxford, Oxford, OX3 9DU, United Kingdom;; ^d^Genome Editing Unit, Faculty of Biology, Medicine and Health, University of Manchester, Manchester, M13 9PT, United Kingdom;; ^e^Target Discovery Institute, Nuffield Department of Medicine, University of Oxford, OX3 7FZ, United Kingdom

**Keywords:** rheumatoid arthritis, inflammation, circadian clock, mitochondria, ceramide

## Abstract

Chronic inflammation underpins many human diseases. Morbidity and mortality associated with chronic inflammation are often mediated through metabolic dysfunction. Inflammatory and metabolic processes vary through circadian time, suggesting an important temporal crosstalk between these systems. Using an established mouse model of rheumatoid arthritis, we show that chronic inflammatory arthritis results in rhythmic joint inflammation and drives major changes in muscle and liver energy metabolism and rhythmic gene expression. Transcriptional and phosphoproteomic analyses revealed alterations in lipid metabolism and mitochondrial function associated with increased EGFR-JAK-STAT3 signaling. Metabolomic analyses confirmed rhythmic metabolic rewiring with impaired β-oxidation and lipid handling and revealed a pronounced shunt toward sphingolipid and ceramide accumulation. The arthritis-related production of ceramides was most pronounced during the day, which is the time of peak inflammation and increased reliance on fatty acid oxidation. Thus, our data demonstrate that localized joint inflammation drives a time-of-day–dependent build-up of bioactive lipid species driven by rhythmic inflammation and altered EGFR-STAT signaling.

## Significance

Rheumatoid arthritis (RA) is a debilitating chronic inflammatory disease in which symptoms exhibit a strong time-of-day rhythmicity. RA is commonly associated with metabolic disturbance and increased incidence of diabetes and cardiovascular disease, yet the mechanisms underlying this metabolic dysregulation remain unclear. Here, we demonstrate that rhythmic inflammation drives reorganization of metabolic programs in distal liver and muscle tissues. Chronic inflammation leads to mitochondrial dysfunction and dysregulation of fatty acid metabolism, including accumulation of inflammation-associated ceramide species in a time-of-day–dependent manner. These findings reveal multiple points for therapeutic intervention centered on the circadian clock, metabolic dysregulation, and inflammatory signaling.

The regular 24-h environmental cycle generated by the planet’s rotation has driven the evolution of intrinsic biological timing mechanisms in virtually all life forms on Earth. In mammals, circadian clocks orchestrate daily rhythms in biology and behavior such that most physiological systems are regulated in a time-of-day–dependent manner (1–3). The core cellular circadian pacemaker in mammals oscillates with a 24-h period and is underpinned by a coordinated feedback between positive transcriptional regulators BMAL1/CLOCK and repressors *Period 1* and *2* (*Per1* and *Per2*, respectively), *Cryptochrome 1* and *2* (*Cry1* and *Cry2*, respectively), and *Nr1d1* and *Nr1d2* (REV-ERBα and REV-ERBβ, respectively) (4). The temporal organization of the reciprocal interactions between these positive and negative arms underpins cellular timekeeping. Beyond this circadian transcription–translation feedback loop, the core clock transcription factors act through clock-controlled genes to regulate multiple aspects of cellular function in a cell- and tissue-specific manner. As prime examples, both energy metabolism and immunity are strongly regulated by the circadian clock, shaping physiological and immune response based on time of day (5, 6). Due to the pervasive nature of the clock within our biology, altered circadian rhythmicity has been recognized as a characteristic feature and/or contributing factor to numerous disease states, with clear implication for diagnosis and treatment (6).

Circadian control of energy metabolism is essential for mounting an appropriate physiological response to recurrent and abrupt changes in nutrient availability, as animals move between wake and sleep states and fed and fasted periods. All metabolic tissues, such as the liver and muscle, have intrinsic circadian oscillators, which are normally synchronized to the external environment and to the internal state by the central pacemaker in the suprachiasmatic nuclei. These peripheral clocks can also be strongly influenced by feeding times, with an important role for nutrient, microbiome, and hormone signaling (3). Indeed, it is now clear that rhythmic control of metabolic circuits exhibits substantial plasticity in response to changing dietary intake, behavioral routine, and/or disease. For example, the hepatic circadian transcriptome is impacted by aging (7) and chronic disease, as in bronchogenic carcinoma, which causes substantial remodeling of circadian hepatic metabolic pathways (8). Moreover, nutritional challenges caused by high-fat diet feeding and subsequent obesity can both directly impact the circadian clock mechanism (9–11) and can substantially rewire rhythmic hepatic circuits (12, 13). In these and other examples, systemic and local inflammatory processes are likely to be driving factors in altered circadian metabolic control.

We previously identified an important role for the circadian clock in shaping immune and inflammatory responses to challenge (14–16); furthermore, inflammatory signals can acutely reset circadian circuits via rapid selective degradation of core proteins of the circadian clockwork (17). In humans, rheumatoid arthritis (RA) is a common, debilitating, chronic inflammatory disease, which can lead to destructive arthritis (18). RA is also prominently associated with accelerated atherosclerosis, elevated cardiovascular risk, and insulin resistance (19, 20), yet the causal mechanisms involved in metabolic disturbance are largely unknown. In mice, collagen-induced arthritis (CIA) has been widely used as a robust model of inflammatory arthritis. Both human arthritis and mouse CIA share striking circadian rhythmicity of joint inflammation, with joint swelling and inflammatory cytokine concentration in peripheral blood varying through circadian time (21, 22). Here, we investigate how chronic joint inflammation (in murine CIA) impacts circadian function, both in the inflamed joint and more distal tissues, and its downstream impact on rhythmic systemic energy metabolism.

Our findings reveal a pronounced impact on rhythmic gene expression within the inflamed joint and also widespread changes to rhythmic transcriptional, phosphoproteomic, and metabolomic profiles in liver and muscle. In these tissues, remodeling of lipid utilization and mitochondrial function were evident, likely driven by increased EGFR and STAT signaling, and culminating in system-wide accumulation of potentially damaging ceramides.

## Results

### Murine Model of Chronic Inflammatory Arthritis Shows Circadian Features.

**Fig. 1.**
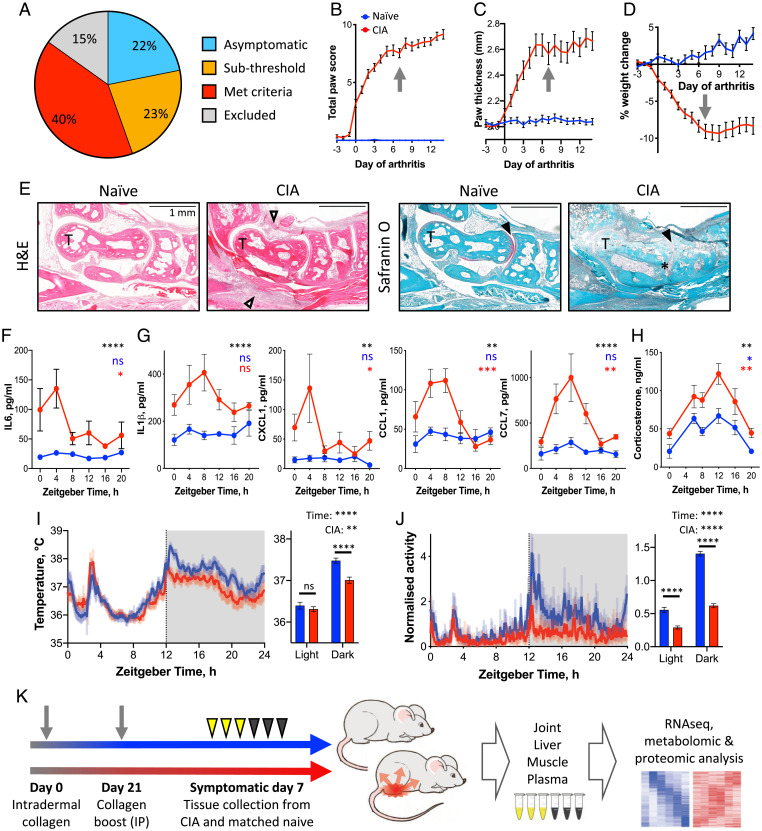
CIA as a model of rhythmic inflammation. (*A*) CIA mice exhibited variation in severity of disease, and were carried forward for subsequent analyses based on selection criteria (*Materials and Methods*). (*B*–*D*) Upon development of symptoms, paw inflammation score (*B*), paw thickness (*C*), and body weight loss (*D*) progressed rapidly before stabilizing at approximately 5 to 7 d after onset (*n* = 14 naïve mice; *n* = 36 CIA mice). Arrows indicate symptomatic day 7, chosen for terminal tissue collection. (*E*) Histological staining of paw sections around the talus (T) from naïve and CIA mice illustrates inflammatory cell infiltration (open arrowheads), loss of cartilage (filled arrowheads), and synovial hyperplasia (*). (*F* and *G*) Plasma cytokine concentrations showed elevation and rhythmicity in arthritic (red) compared with naïve mice (blue) (treatment comparison by two-way ANOVA indicated in black; adjusted *P* value of JTK analysis indicated in blue [naïve mice] and red [CIA mice]; *n* = 4–5/point). (*H*) Plasma corticosterone level was elevated and rhythmic in CIA mice (treatment comparison by two-way repeated-measures ANOVA in black; rhythmicity, according to Lomb-Scargle analysis, is indicated in blue (naïve mice) and red (CIA mice), *n* = 5–9/condition). (*I* and *J*) Body temperature (*I*) and activity (*J*) similarly showed maintained rhythmicity in CIA mice (3-d average recordings; blue, presymptomatic; red, symptomatic days 5–7). Summary statistics exclude ZT2–3.5, when the mice were handled for scoring (two-way repeated-measures ANOVA in black; *n* = 7–8). (*K*) Schematic showing experimental design. Data are presented as mean ± SEM throughout; details of statistical tests are provided in Dataset S1. *P < 0.05, **P < 0.01, ***P < 0.001, ****P < 0.0001. IP, intraperitoneal; RNAseq, RNA sequencing.

To investigate how chronic inflammatory disease impacts systemic energy balance and circadian function in local (joint) and distal (liver and muscle) sites, we used the CIA model in DBA/1 mice. To increase consistency between animals, we included for further analyses only mice which achieved a total paw score ≥6 involving at least two limbs (Fig. 1*A*; see *SI Appendix* for experimental details). Disease in affected mice increased rapidly from the emergence of symptoms before reaching a chronic and relatively stable inflammatory state around 5 d after onset (Fig. 1 *B* and *C*). Body weight and fat-mass loss in the animals was mild and followed a similar trajectory (Fig. 1*D* and *SI Appendix*, Fig. S1*A*). Arthritic joints were characterized by inflammatory cell infiltration, cartilage erosion, and proliferation of the synovium (Fig. 1*E*), mimicking disease processes evident in patients with RA (23).

We previously demonstrated that disease symptoms in CIA mice exhibit diurnal rhythmicity, with exacerbation of joint inflammation observed in the day (21). This was similarly reflected in the present study in circulating inflammatory cytokine and chemokine levels (Fig. 1 *F* and *G*), with peak plasma levels occurring during the daytime (the animals’ typical rest phase; approximately zeitgeber time [ZT] 4–8). Importantly, robust behavioral and physiological rhythms were maintained in the CIA-affected mice, as demonstrated in profiles of plasma corticosterone (Fig. 1*H*), body temperature (Fig. 1*I*), and food intake (*SI Appendix*, Fig. S1 *B*–*D*). We saw no significant change in fasting plasma glucose levels, although fasting insulin levels were reduced in CIA mice (*SI Appendix*, Fig. S1 *E* and *F*). Total locomotor activity was reduced in CIA mice relative to presymptomatic levels yet retained a significant diurnal variation (Fig. 1*J*).

### Inflamed Joint Shows Extensive Changes in Circadian Transcriptional Regulation.

**Fig. 2.**
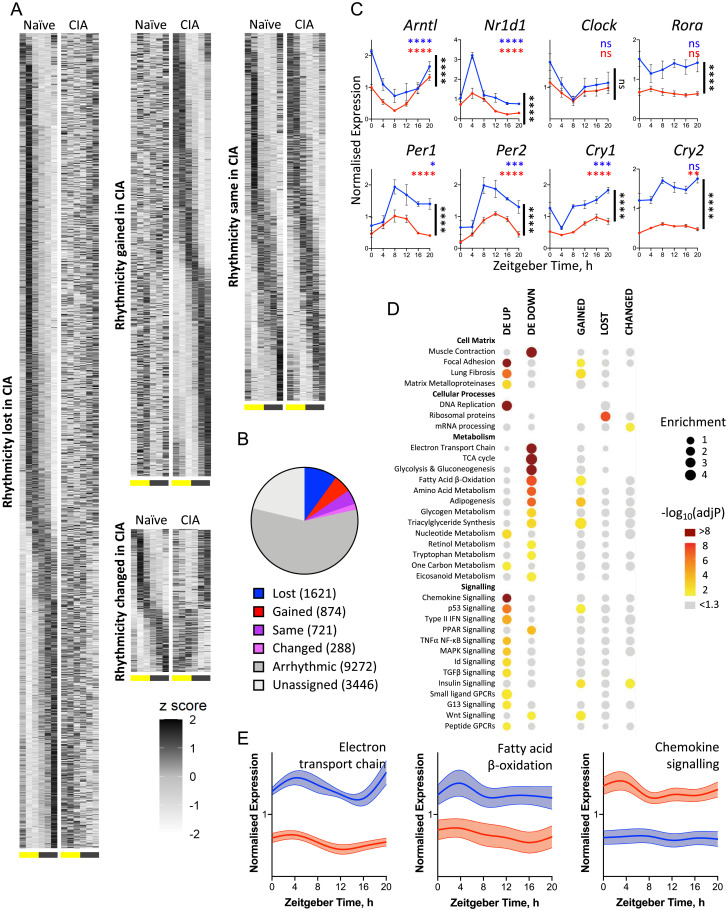
Inflamed joint tissue shows extensive changes in circadian transcriptional regulation. (*A* and *B*) Joint samples from naïve and CIA mice were collected on symptomatic day 7 at six time points and analyzed by RNA sequencing (*n* = 5/time point per condition). Heat maps (*A*) represent the normalized (*z*-scored) transcript expression levels in naïve (*Left*) and CIA (*Right*) mice over time (columns, from ZT0 at 4-h intervals). Differential rhythmicity analysis categorized transcript expression profiles according to change or maintenance of rhythmicity with disease (*B*). (*C*) CIA significantly altered the transcript expression of a number of core clock genes (CIA vs. naïve comparison by two-way ANOVA indicated in black; adjusted *P* [adjP] value of JTK analysis indicated in blue and red; *n* = 5/point. *P < 0.05, **P < 0.01, ***P < 0.001, ****P < 0.0001. Data are presented as mean ± SEM). (*D*) Groups of genes showing significant differential expression (DE) or rhythmic change with disease were analyzed for functional enrichment using the Enrichr tool (*Materials and Methods*) to identify altered gene expression across metabolic and signaling pathways (spot size represents fold enrichment of genes in group vs. the genome; color represents significance of enrichment; spot absence means no genes from the pathway were allocated to the group on statistical categorization). (*E*) Spline graphs show the mean normalized expression of all genes in selected WikiPathways gene sets (WP295, WP1269, and WP2292; error bars represent 95% CIs around the mean), demonstrating altered pathway expression in CIA mice. GPCR, G-protein-coupled receptor; ns, not significant.

To characterize the impact of active inflammatory joint disease on circadian and metabolic processes, joint, liver, and muscle tissues, as well as blood plasma, were collected every 4 h across the diurnal cycle (*n* = 5 per time point per condition), 7 d after symptom onset in CIA mice and time-matched controls (Fig. 1*K*; weight and disease scoring for these mice is shown in *SI Appendix*, Fig. S1 *G*–*I*). For assessment of transcriptional rhythmicity in naïve and CIA mice, we used a robust comparative approach (compareRhythms) based on probability assignment to arrhythmic, matched (“same”), or differential (“gained,” “lost,” “changed”) rhythmicity between the two conditions [Fig. 2 *A* and *B* (24)]. This contrasts with conventional analyses in which rhythmicity of gene expression is defined separately for each condition [e.g., using the JTK-cycle algorithm (25)], and differences between conditions are inferred based on overlapping gene lists (included for comparison in *SI Appendix*, Fig. S2*A*). Significant benefits to our approach are that analysis is more conservative, avoiding false discovery, and that we identify genes which show altered rhythmic expression profiles between the conditions. Within the diseased joint, we found genome-wide changes in the circadian organization of gene expression (Fig. 2 *A* and *B*). Across all conditions, 3,504 genes showed high confidence rhythmicity, of which almost 80% either lost, gained, or showed a change in rhythmic profile with disease. The change in rhythmic gene expression within the inflamed joint suggested a profound alteration in clock control of cell and tissue processes. Indeed, profiling of core circadian clock components themselves revealed that, while rhythmic expression was widely maintained, significant reduction in overall expression and amplitude was evident (Fig. 2*C*).

Pathway enrichment analyses on genes which showed loss of rhythmicity between naïve and inflamed joints only found significant enrichment for transcripts associated with ribosomal proteins (Fig. 2*D*). Ribosome maturation, mRNA translation, and protein turnover are all known to show time-of-day rhythmicity (26). Interestingly, genes that became rhythmic under CIA showed significant enrichment of metabolic targets involved in lipid metabolism and β-oxidation. These newly rhythmic genes included enzymatic regulators of triglyceride and fatty acid metabolism (e.g., *Lpl*, *Lipe*, *Plin2*, *Lipin1/3*, *Cpt1a*), as well as influential signaling and transcriptional regulators (e.g., *Nr3c1*, *Ppard*, *Stat3*, *Socs3*) (*SI Appendix*, Fig. S2*B*).

We also examined the impact of disease state (naïve vs. inflamed joints) on gene expression irrespective of time of day (*n* = 30 mice/condition). To achieve a robust comparison, we used a strict false discovery rate (FDR) cutoff (<1 × 10^−20^) and identified a highly significant set of 5,649 differentially expressed (DE) genes. As expected for inflammatory joint disease, pathways enriched with up-regulated genes included inflammatory and cell-matrix remodeling processes, while down-regulated genes were associated with metabolic pathways (Fig. 2 *D* and *E*).

Given the widespread alteration of gene expression within the inflamed joint and the notable emergence of newly rhythmic genes, we next examined potential upstream regulators using Ingenuity Pathway Analysis. Analyses of the DE gene set implicated several known inflammatory mediators (namely, IκBα, STAT1, STAT3, STAT6, NR4A1), as well as epigenetic (KDM5A, SMYD1) and metabolic (PPARγ, PGC1α, PGC1β) regulators (*SI Appendix*, Fig. S2*C*). Upstream regulator analyses based on genes which showed altered rhythmicity in the arthritic joint identified transcription factors previously shown to be capable of driving rhythms in gene expression, including PPARγ, PGC1α, GR (NR3C1), SIRT1, and HIF1α (*SI Appendix*, Fig. S2*C*).

Thus, within the inflamed joint, there is clear disturbance of the molecular clockwork and associated and pronounced alteration in the rhythmic transcriptome. This altered activity with disease reflects the changing metabolic and inflammatory states and suggests altered dominance of nonclock regulators (e.g., PPARs, GR) in driving time-of-day differences in gene expression.

### Inflammatory Arthritis Reprograms Gene Expression in Muscle and Liver.

**Fig. 3.**
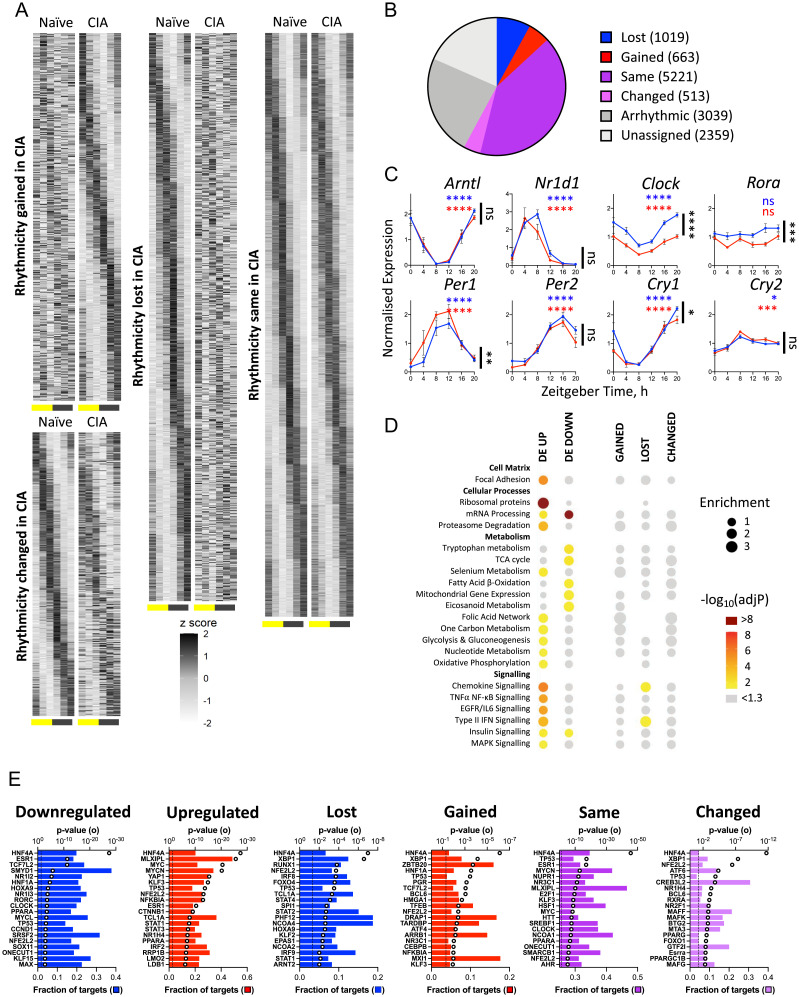
The liver transcriptome shows circadian perturbation in response to arthritis. (*A* and *B*) Matched liver samples were analyzed by RNA sequencing to characterize the effect of distal inflammatory disease. The majority of genes were rhythmic under at least one condition, and genes were grouped according to change or maintenance of rhythmicity with CIA using compareRhythms (Fig. 2). (*C*) Rhythmic expression of most core clock genes was maintained in liver, although expression of *Clock* and *Rora* was significantly reduced with disease (CIA vs. naïve comparison by two-way ANOVA in black; adjusted *P* [adjP] value of JTK analysis in blue and red; *n* = 5/point. *P < 0.05, **P < 0.01, ***P < 0.001, ****P < 0.0001. Data are presented as mean ± SEM). (*D*) Groups of genes showing significant differential expression (DE) or rhythmic change with disease were analyzed using the Enrichr tool (*Materials and Methods* and Fig. 2). Enrichment of metabolic and signaling categories were predominantly associated with changes in expression level rather than rhythmicity. (*E*) Upstream regulator analysis of liver transcripts differentially expressed with CIA identified metabolic and inflammatory mediators. The top 20 most significant regulators are shown for each category. Open circle represents significance of enrichment; filled bar represents fraction of downstream targets found in the category; dashed line indicates *P* = 0.05. ns, not significant.

To define how localized joint inflammation may impact metabolic and rhythmic processes at distal sites, we next profiled liver and muscle. Analyses of liver (Fig. 3 *A* and *B*) and muscle (*SI Appendix*, Fig. S3 *A* and *B*) tissue isolated from naïve or CIA mice showed that rhythms in gene expression were widely maintained, although ∼30% of oscillatory genes showed altered rhythmicity (loss, gain, or change) between naïve and CIA animals. Employing standard JTK-cycle analyses with a subsequent overlap comparison identified many more genes which potentially gained or lost significant rhythmicity (*SI Appendix*, Fig. S4 *A* and *B*). However, as this approach does not directly compare individual gene expression profiles, it is prone to high FDRs (*SI Appendix*, Fig. S4*C*).

Core circadian clock genes continued to oscillate robustly in the liver (Fig. 3*C*) and muscle (*SI Appendix*, Fig. S3C) of CIA mice, although *Clock* and *Rora* expressions were significantly reduced in CIA liver, and other minor alterations were observed. Thus, compared with the joint, disruption to core clock gene expression in liver and muscle appeared subtle, which would be expected when comparing the primary site of inflammation with more distal tissues. Pathway enrichment analyses of differentially rhythmic genes in liver showed few significantly enriched processes (only chemokine and IFN signaling; Fig. 3*D*). Similarly, in muscle, only circadian regulation emerged as enriched in the “changed” group (*SI Appendix*, Fig. S3*D*).

In contrast, CIA had a widespread impact on gene expression when examined irrespective of time (*n* = 6,172 DE genes, FDR < 0.001 in liver; *n* = 2,800 DE genes, FDR < 0.001 in muscle). Pathway enrichment analyses of DE genes in the liver revealed a number of highly enriched pathways, which were suggestive of altered protein dynamics (ribosome proteins, proteasome degradation), mitochondrial and lipid metabolism (tricarboxylic acid cycle [TCA], β-oxidation, mitochondrial gene expression), and increased inflammatory signaling (Fig. 3*D*). Upstream regulator analysis identified MLXIPL as a candidate regulator of DE genes in the liver (Fig. 3*E*). MLXIPL has been found to drive fatty acid and triglyceride production, activated by a glucose metabolite (27). The prominence of HNF4 as a predicted upstream regulator is likely to reflect its widespread influence over hepatic gene expression rather than specific perturbation of HNF4 activity during CIA. Inflammatory mediators were highlighted (namely, STAT1, STAT3, IκBα). Interestingly, circadian clock factors (CLOCK, RORγ) and a known clock-associated factor (PPARα) were also identified, suggesting that altered clock function may contribute to altered gene regulation during CIA beyond dictating rhythmicity.

Pathway enrichment analyses of DE genes in muscle paralleled that observed in liver, with ribosomal proteins, proteasome degradation, inflammatory signaling, and mitochondrial metabolism widely represented (*SI Appendix*, Fig. S3*D*). Moreover, significantly altered expression of key metabolic regulators, including *Ppar*s, *Pgc1a*, *Mlxipl*, and components of the JAK-STAT signaling pathway were evident in CIA muscle (*SI Appendix*, Fig. S3*E*). Upstream regulator analysis of the muscle DE gene sets identified similar transcription factors involved in metabolic (e.g., PGC1α, PGC1β, MLXIPL) and inflammatory (e.g., STAT3, RELA, IκBα, NR4A1) function, as well as known clock and stress-responsive factors HIF1α and SIRT1 (*SI Appendix*, Fig. S3*F*). These findings suggest that CIA leads to a state of increased inflammatory signaling and cell stress within both tissues, with consequent impact on lipid and mitochondrial metabolism.

### Identification of EGFR and STAT as Mediators of Hepatic Response to CIA.

**Fig. 4.**
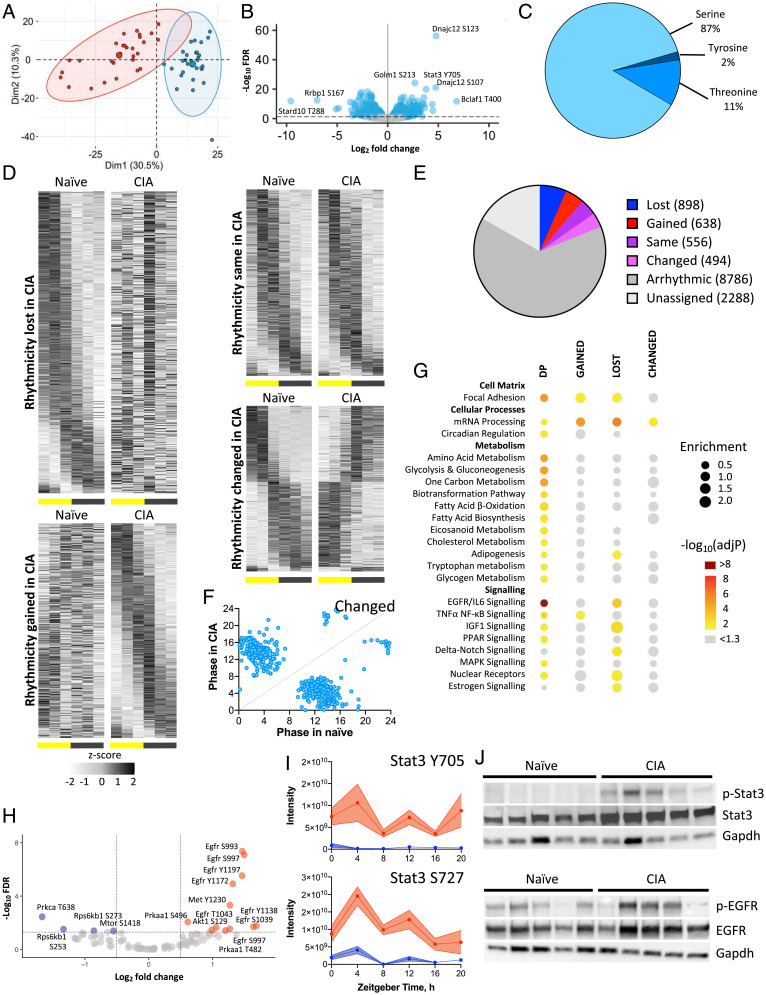
Phosphoproteomic analysis of liver identifies EGFR signaling as a potential mediator of inflammatory disease response. (*A*–*C*) The phosphoproteome of the same liver samples (*n* = 5/time per condition) was analyzed by mass spectrometry. Principle component analysis identified disease as the largest source of variation (*A*; naïve mice, blue; CIA mice, red). Significantly altered phosphopeptides (*B*, blue), predominantly serine residues (*C*), were identified. (*D*–*F*) Differential rhythmicity analysis using compareRhythms (*D* and *E*) highlighting a switch in predicted phase for phosphopeptides belonging to the “altered” group (*F*). (*G*) Groups of genes associated with sites of DP and/or rhythmic phosphorylation were analyzed for functional enrichment using the Enrichr tool (spot size represents fold enrichment of genes in group vs. the genome; color represents significance of enrichment; spot absence means no genes from the pathway belonged to the group), highlighting enrichment of genes involved in EGFR/IL6 signaling. (*H*) The KinSwingR package was used to predict kinases associated with significant (FDR < 0.05) phosphoproteome changes (increased phosphorylation with CIA indicated in red; reduced phosphorylation indicated in blue), also highlighting EGFR signaling. (*I*) Among predicted regulatory kinases, Stat3 showed DP. Treatment effect by two-way ANOVA. ****P < 0.0001. Error bars represent SEM. (*J*) Western blot analysis of phospho-Stat3, Stat3 (*Top*), phospho-EGFR and EGFR (*Bottom*) confirmed increased phosphorylation in CIA liver samples collected at ZT4. GAPDH immunoblot was used as a loading control.

Transcriptional analyses implicated a number of signaling cascades with potential to drive altered tissue and metabolic function in the liver and muscle during CIA. However, many of these cascades are regulated posttranslationally; therefore, we next examined disease-related changes to the hepatic phosphoproteome across the diurnal cycle. From a total of 13,660 phosphorylation events identified, 1,826 (13.4%) were significantly altered on comparison of CIA and naïve conditions (Benjamini-Hochberg FDR < 0.05; Fig. 4 *A*–*C*).

Approximately 25% of detected phosphopeptides were significantly rhythmic, and comparison between naïve and disease states revealed widespread differential rhythmicity (Fig. 4 *D* and *E*). Notably, species identified as having an altered rhythmic profile between the two conditions (i.e., changed) exhibited a dramatic shift in peak phase (Fig. 4*F*). Enrichment analysis of genes corresponding to significantly altered phosphopeptides, either as a result of differential phosphorylation (DP) with disease or changes in rhythmicity, identified pathways involved in proinflammatory signaling (including EGFR1/IL-6 and NFκB) and metabolic (amino acid and glucose metabolism, β-oxidation, PPAR regulated) processes (Fig. 4*G*). Disease-related alteration in phosphorylation state rhythmicity was observed in influential regulators of energy metabolism (e.g., AKT, IR, IRS1 and 2, mTOR, SREBF1, FOXO1 and 3, SIRT1, CPT1a, MLXIPL). These findings closely parallel outputs from transcriptional and upstream regulator analyses of naïve and CIA liver and reveal a pronounced impact of joint inflammation on hepatic energy handling.

To identify upstream kinases likely to contribute to the DP profiles observed in naïve versus CIA liver, we next used the KinSwingR algorithm (28) and the PhosphoSitePlus database. Forty-nine candidates were identified based on kinase-substrate interaction predictions (*SI Appendix*, Table S1). Of the predicted kinases, 29 were directly identified within our dataset, and seven of these showed DP states between naïve and CIA conditions (FDR < 0.05; Fig. 4*H*). EGFR, AMP-kinase, and AKT showed increased phosphorylation with CIA, while mTOR and its associated mediator, ribosomal P70-S6 kinase, had reduced phosphorylation. Among other significantly phosphorylated proteins, STAT3 had increased phosphorylation at both Y705 and S727 (FDR < 0.05; Fig. 4 *B* and *I*). Both sites on STAT3 had significantly increased phosphorylation across the time series, and S727 phosphorylation also showed significant rhythmicity with CIA. These STAT3 phosphorylation sites are known to promote nuclear translocation, DNA binding, and transcriptional regulation of widespread gene targets, which, in the liver, include influential regulators of energy metabolism (29). In line with increased STAT3 activity, expression of its well-characterized target gene, *Socs3*, significantly increased in the livers of CIA mice, as did expression of *Stat3* itself (*SI Appendix*, Fig. S5*A*).

In CIA, multiple signaling pathways appeared to converge on the JAK-STAT cascade, including EGFR activation and the inflammatory cytokines IL6 and IL1β. In line with phosphoproteome profiling, increased total and phosphorylated forms of EGFR and STAT3 were observed in livers recovered from the CIA mice compared with those from naïve animals (Fig. 4*J*). Thus, our phosphoproteomics reinforces the CIA-driven alteration in signaling response with downstream metabolic consequences. Supporting a prominent role for liver STAT3 action in the CIA mice, we find a high proportion of genes that are differentially regulated by CIA to be associated with STAT3-binding sites in hepatocytes treated with IL6 (*SI Appendix*, Fig. S5*B* (30)). These targets are functionally enriched for genes involved in fatty acid β-oxidation and mitochondrial function, as well as canonical targets of inflammatory signaling pathways (*SI Appendix*, Fig. S5*C*).

### Metabolomic Profiling Highlights Differential Lipid Use in Distal Tissues.

**Fig. 5.**
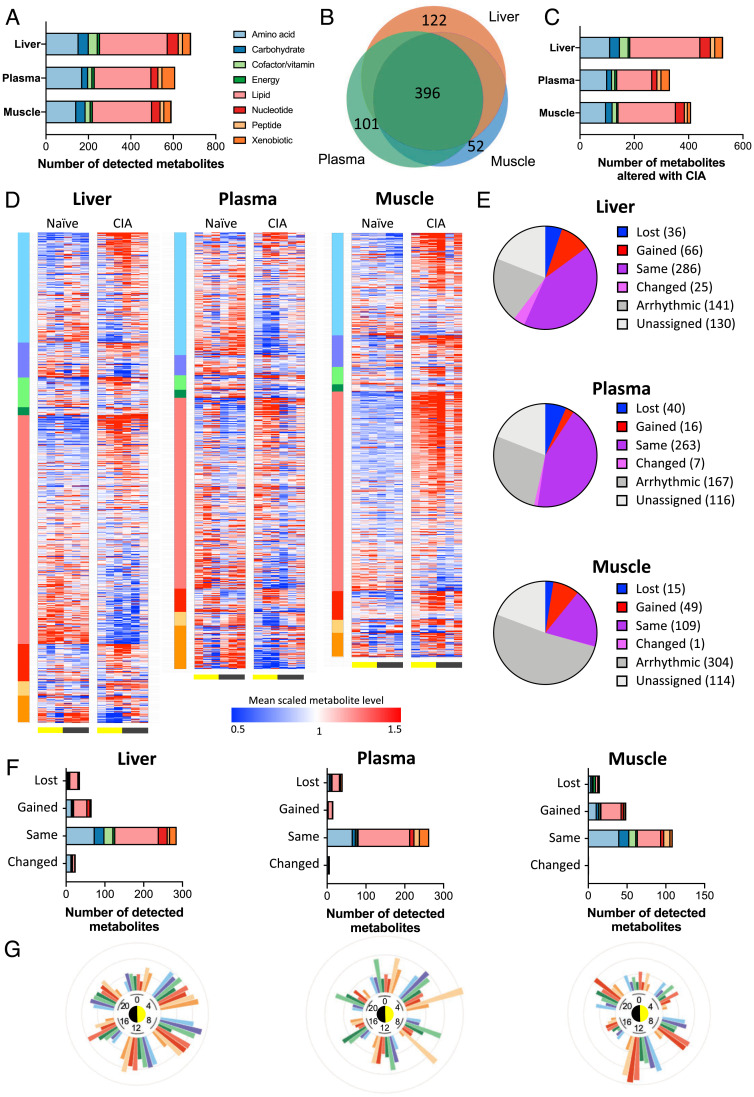
The majority of detected metabolites in distal tissues are significantly altered by inflammatory disease. (*A* and *B*) Global metabolomic profiling of samples from naïve and CIA mice (*n* = 5/time per condition) identified overlapping metabolite profiles in liver, plasma, and muscle. Color indicates metabolite superclass of detected metabolites; scale indicates number of identified metabolites. (*C*) Significant numbers of detected metabolites showed differential abundance with disease (*q* < 0.05 on two-way ANOVA). (*D*) Heat maps of tissue-specific metabolite abundance (sorted by metabolite class and then fold change in detection level) represent alterations to metabolite level with disease. (*E* and *F*) The effect of distal inflammatory disease on metabolite rhythmicity was categorized using compareRhythms (*E*) and represented by superclass (*F*). (*G*) The proportion of metabolites showing differential detection between naïve and CIA tissue at each time point are presented on radial plots, indicating tissue- and time-specific changes in metabolite abundance with disease.

Analysis of gene expression in liver and muscle identified lipid and mitochondrial metabolism to be differentially regulated by CIA, while phosphoproteomic analysis confirmed changes in EGFR and STAT3 signaling as potential upstream regulators of altered metabolic function in the liver. We next assessed the consequences of CIA through metabolomic profiling of plasma, muscle, and liver samples from the same naïve and CIA-affected mice. The number and class distributions of detected metabolites were comparable to those reported from previous studies [e.g., Dyar et al. (13)] with more than 850 unique species quantified and ∼30% of species showing tissue-specific detection (Fig. 5 *A* and *B*).

We first examined metabolite levels irrespective of time of day. CIA caused a pronounced and system-wide alteration in metabolites between naïve and arthritic mice (with 77%, 59%, and 69% of detected metabolites showing a significant difference in abundance in response to disease in liver, plasma, and muscle, respectively; *q* < 0.05; Fig. 5 *C* and *D* and *SI Appendix*, Fig. S6*A*). The impact of CIA was evident across metabolite superfamily classifications (Fig. 5*C*). Widespread rhythmicity was observed in ∼30% of muscle and ∼55 to 60% of liver and plasma metabolites (Fig. 5 *D* and *E*). Again, this was reflected across metabolite classes (Fig. 5*F*). Comparison of disease-related change (irrespective of time) in specific metabolites across tissues revealed a generally coordinated response between liver and muscle, except for a notable divergence observed in lipid species (which were increased in muscle, but reduced in liver in CIA mice relative to naïve mice; *SI Appendix*, Fig. S6 *B* and *C*).

**Fig. 6.**
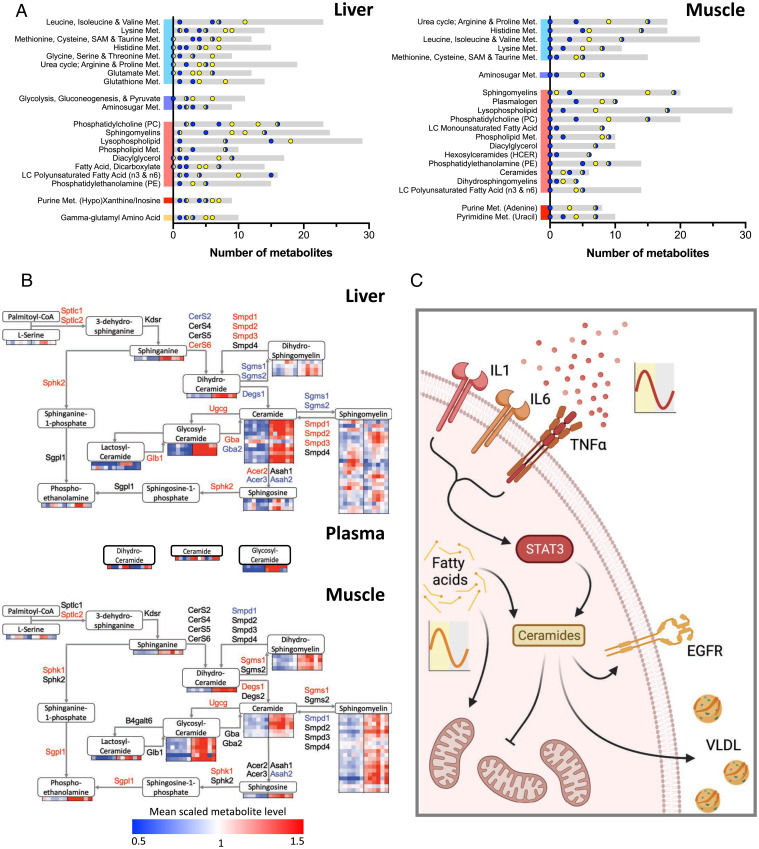
Metabolomic profiling highlights differential lipid use in liver and muscle. (*A*) Metabolite subpathways were sorted according to average differential detection between light (ZT4, 8, 12) and dark (ZT16, 20, 0) conditions to identify metabolic processes altered by disease and diurnal rhythm in liver (*Left*) and muscle (*Right*) (gray bars represent total detected metabolites; points represent number of differentially detected metabolites at light (yellow), dark (blue), or transition (yellow and blue) time points). (*B*) Integrating differential gene expression (named genes; red and blue indicate significant up- and down-regulation, respectively) and metabolite-detection profiles (heat maps) into a schematic representation of the ceramide biosynthesis pathway in liver (*Top*) and muscle (*Bottom*) highlights the accumulation of ceramides and sphingolipids with disease. (*C*) We propose a model in which chronic inflammatory signaling impacts rhythmic metabolism in distal tissues via the EGFR/STAT3 circuit. This results in rhythmic accumulation of fatty acid precursors, increased ceramide abundance, and deleterious effects upon mitochondrial function. Fig. 6*C* was created with BioRender.com. Met, metabolism.

Individual metabolite rhythmicity was largely maintained in the tissues and plasma (relatively few were classified as “loss”). However, there was a pronounced emergence of oscillating metabolites in both liver and muscle of CIA mice, which was particularly evident in lipid species (Fig. 5*F*). Moreover, temporal correlation between metabolites (both within and across tissues) was significantly reduced in CIA mice when compared with naïve controls (*SI Appendix*, Fig. S6*D*). By comparing the proportion of altered metabolites (between naïve and CIA samples) within each superclass by time of day, we observed that the impact of CIA increased during the mid to late day (ZT4–12; Fig. 5*G*). Extending this analysis to metabolite subclasses, we found that a number of influential lipid subclasses, including ceramides, phospholipids, long-chain fatty acids (LCFAs), and acyl carnitines, showed an overall and time-related response to CIA (Fig. 6 *A* and *B* and *SI Appendix*, Fig. S7*A*). Thus, while the effects of CIA were observed across all metabolite classes (in terms of level and rhythmicity), the effect upon lipid species was pronounced, especially during the mid to late day (Fig. 5 *F* and *G*). This is a time when disease activity and circulating cytokines are increased (Fig. 1 *F*–*H*) and when the animals have an increased reliance on fatty acid oxidation for energy (due to the natural rest and activity cycle of the nocturnal mice).

Transcriptional profiling in both muscle and liver implicated an alteration in mitochondrial metabolism (electron transport chain, TCA, and β-oxidation pathways) in CIA. Although complex, altered mitochondrial activity and lipid metabolism were also reflected in metabolomic profiles (*SI Appendix*, Fig. S7*B*). For example, liver showed a significant decrease in LCFAs but time-of-day–dependent accumulation of other lipid species, including long-chain acyl carnitines, β-hydroxybutyrate, and endocannabinoids. A particularly notable shift in hepatic and muscle lipid profile was observed in the ceramides, phosphatidylcholines, and sphingomyelins (Fig. 6*B* and *SI Appendix*, Fig. S7*C*). This suggests that inefficiency in β-oxidation and/or downstream TCA and respiratory chain function in the liver may shunt fatty acids into alternative metabolic pathways, which is accentuated during the day by increased reliance on fatty acid β-oxidation. In muscle, there was an accumulation of lipid metabolites in CIA, which included LCFAs, sphingomyelins, and ceramides (*SI Appendix*, Fig. S7*C*). Here, uptake of liver-produced lipids and ketones is likely.

Ceramide concentrations were significantly higher in both liver and muscle of CIA mice (Fig. 6*B*). Ceramide production from membrane-integral sphingomyelins is a well-characterized, early step following IL-1β/TNFα receptor activation (31). Ceramides can serve as important signaling mediators, affecting cellular energy metabolism but also driving cell stress, metabolic dysfunction, and insulin resistance (31, 32). Analysis of gene expression and intermediary metabolites across the ceramide synthesis pathway in liver supports the shunt into increased ceramide accumulation in CIA mice (Fig. 6*B*, *Top*). We found increased expression of three *Smpd* isoforms (*SI Appendix*, Fig. S8*A*), which catalyze the conversion of complex sphingomyelins to ceramides via the SMase pathway (Fig. 6*B*), as well as up-regulation of the *de novo* synthesis pathway (*Sptlc* isoforms, *CerS6*) and down-regulation of enzymes which convert ceramides to sphingosines (*Acer3, Asah2*). Inflammatory challenge in vivo via systemic administration of lipopolysaccharide increased *Stat3* and *CerS6* expression in liver but did not result in significantly altered expression of other genes involved in ceramide synthesis and metabolism (*SI Appendix*, Fig. S8*B*). The phenotype of ceramide accumulation we observe with CIA may therefore be a result of chronic inflammatory signaling and tissue adaptation rather than responses to acute challenge. Ceramide accumulation in CIA muscle was also clear, but differential gene expression suggestive of increased local production was less evident in this tissue (Fig. 6*B*, *Bottom*). Accumulation may therefore indicate export of ceramides from the liver (e.g., via very-low-density lipoprotein [VLDL] secretion) and subsequent uptake into muscle in CIA mice. In line with this, levels of plasma glycosyl/hexosylceramide species (the only plasma-borne ceramides detected) were significantly elevated in the CIA mice (Fig. 6*B* and *SI Appendix*, Fig. S7*D*).

Overall, our findings reveal a widespread impact of CIA with systemic and local inflammation driving mitochondrial inefficiency and accumulation of damaging lipid species, including ceramides and phosphatidylcholines. While clock function and rhythmic gene expression was broadly maintained, a strong time-of-day impact was observed, likely driven by the convergence of increased inflammation (e.g., circulating inflammatory cytokines) and the increased mobilization of fatty acids for energy supply during the late day.

## Discussion

Energy metabolism and immunity both show strong circadian variation, and abundant evidence supports reciprocal regulation. Metabolic disease in states of chronic inflammation is known to be an important driver of morbidity and mortality, yet little is known of how chronic inflammation impacts on rhythmic energy metabolism and its control circuits. Here, we show that CIA drives a strongly rhythmic systemic inflammatory state with consequent impact on energy metabolism in liver and muscle (Fig. 6*C*).

Overall, we observed significant changes in rhythmicity in the joint, including within the circadian clockwork. In liver and muscle, we saw newly rhythmic or enhanced rhythmicity within transcriptional, metabolomic, and phosphoproteomic data but no changes in circadian clock rhythmicity. Our data suggest that this is due to the convergence of a rhythmic inflammatory state with natural day-to-night changes in physiology (i.e., switch from a fasted to fed state), as well as engagement of regulators capable of driving rhythmic cell function (e.g., PPARs, GR). Similar emergence of transcriptional and metabolite rhythmicity in the liver has been detailed in response to high-fat diet feeding (13, 33).

The emergence of rhythmic inflammatory circuits within experimental chronic inflammatory arthritis offers an attractive model for the prevalent, and also rhythmic, human RA. Despite being distal to the site of inflammation, both liver and muscle showed differential expression of large numbers of genes, with emergence of MLXIPL as a candidate upstream regulator. MLXIPL is a master regulator of fatty acid synthesis, modulated by insulin signaling and EGFR activation (34). Genes with persisting circadian rhythmicity were significantly enriched for core clock targets, while newly rhythmic liver genes in CIA were predicted to be targets of inflammatory (NFKBIA, CEBPB) and glucocorticoid (NR3C1/GR) signaling regulators as well as TFEB, a master regulator of lysosome biogenesis and autophagy, potentially reflecting energy depletion. Simultaneous activation of anabolic (EGFR) and catabolic (TFEB) signals in liver may explain the complex tissue response to chronic inflammation (Fig. 6*C*), and we see up-regulation of the inflammatory NFκB signaling pathway in all tissues. Clear changes in the liver phosphoproteome with CIA enabled us to identify several kinase-substrate pairs, among which the EGFR-STAT3 circuit was prominent, consistent with our transcriptomic analysis. This suggests a gain in EGFR action may be responsible for driving adaptive changes in gene expression, resulting in altered cellular energy homeostasis in liver.

In muscle, lipid metabolites show strong rhythmicity and generally increased levels with CIA, while many, including LCFAs and sphingomyelin species, were decreased in liver (Fig. 6). We observed loss of fat mass in arthritic mice (*SI Appendix*, Fig. S1*A*), suggesting increased adipose lipolysis. The reduction in liver LCFAs, and reciprocal increase in muscle accumulation, implies trafficking by exported VLDL and low-density lipoprotein (35). These lipoprotein moieties carry sphingomyelins and ceramides, which might explain the accumulation of these important membrane lipids in muscle (Fig. 6*B*). The metabolic response of liver to CIA thus impacts system-wide lipid distribution and metabolism. Reduced ambulatory activity in arthritic mice, with the attendant reduction in fatty acid β-oxidation in muscle mitochondria, might also contribute to accumulation of LCFAs in this tissue.

Interestingly, ceramide accumulation in liver and muscle would be expected to cause EGFR activation through action in the plasma membrane (34, 36). EGFR, via PI3kinase signaling, AKT activation, and phosphodiesterase 3B–dependent cyclic adenosine monophosphate degradation, inhibits the catabolic signaling cascade initiated by hormones, including glucagon and adrenaline (37). The resulting production of fatty acids increases esterification and triglyceride secretion in VLDL and also the production of complex fatty acid derivatives, including ceramides and sphingomyelins. We previously identified increased and newly rhythmic circulating ceramide levels in patients with RA (38). Here, we believe this constitutes a reinforcing loop with the predicted effect of exacerbating the impact of oscillating levels of inflammatory cytokines on liver metabolism. The change from an oscillating input signal (cytokine concentration) to a stable signaling output (up-regulation of STAT3 signaling and ceramide abundance) offers a potential mechanism to explain the metabolic dysfunction associated with human chronic inflammatory disease within the context of circadian regulation, including activation of signaling cascades typically associated with both anabolic and catabolic programs and defects in the mitochondrial electron transport chain (39) (Fig. 6*C*).

It is important to acknowledge potential study limitations. Characterizing true rhythmicity in biological factors is complex, with numerous study designs and analysis approaches available. We used a comparative analytical approach (24), which is highly appropriate for comparing rhythmicity between conditions. Nevertheless, all determination of rhythmicity is based on statistical threshold and/or probability and, therefore, cannot be quantified in absolute terms. For this reason, we have focused on pathway- and process-level changes to guide our interpretations, as well as on drawing insight across biological levels (i.e., transcript, phosphoprotein, and metabolite). In addition, while the CIA model provides a robust experimental model of RA, there are, of course, many differences between the mouse and human disease, including chronicity of the inflammatory process. Even in mice this is a complex disease which shows variability between individuals and leads to additional behavioral effects (e.g., reduced locomotor activity). To mitigate this, we implemented strict inclusion criteria regarding disease severity and collected robust numbers of replicate samples over multiple independent experimental studies (for fuller discussion see *SI Appendix*, *Supplemental Discussion and Study Limitations*).

Overall, we have identified system-wide circadian rewiring resulting in adaptive changes in energy metabolism with chronic inflammation. Prominent players driving this change include circulating inflammatory cytokines, including IL-1β and IL6, which drive response pathways, including EGFR-STAT3, that result in changes to lipid synthesis, transformation, modification, and β-oxidation. Liver accumulation of long-lived ceramide species, driven by increased *CerS6* (as seen in our model), affects both plasma membrane and mitochondrial membrane function, negatively impacting efficient TCA function, and may also result in export to peripheral tissues, including muscle, by VLDL transport (40). The time-of-day–dependent accumulation of fatty acid precursors expedites sphingomyelin and ceramide synthesis, perpetuating this deleterious process and the distal metabolic effects of chronic inflammation.

## Materials and Methods

### Animals.

Mice were maintained in the University of Manchester Biological Services Facility. All protocols were approved by the University of Manchester Animal Welfare and Ethical Review Body and carried out according to the Animals (Scientific Procedures) Act 1986.

### CIA.

The CIA model was applied to male DBA/1 mice (aged 8–10 wk) as described by Hand et al. (21) and in *SI Appendix*, *Extended Materials and Methods*. Tissue collection occurred at ZT0, 4, 8, 12, 16, or 20 on day 7 after the development of disease symptoms in CIA mice, or at matched time points in naïve mice. Body composition was measured using an EchoMRI E26-258-MT machine (Echo Medical Systems). Activity and body temperature measurements were made following surgical implantation of radio telemetry devices (TA-F10, Data Sciences International) intraperitoneally 7 to 10 d after initial CIA immunization. Disease characterization is described in *SI Appendix*, *Extended Materials and Methods*.

### RNA Analysis.

RNA was extracted from joint, liver, and muscle tissues from naïve and CIA mice. Sequencing libraries were generated using the TruSeq Stranded mRNA assay (Illumina, Inc.) and analyzed by paired-end sequencing on a HiSEq 4000 instrument. Differential expression analysis was run in R (41) using edgeR (version 3.30.3). Differential rhythmicity analysis was performed using the compareRhythms R package [version 0.99.0 (24)]. Pathway analysis of differentially expressed and rhythmic genes used the Enrichr web tool (42, 43). Additional details are provided in *SI Appendix*, *Extended Materials and Methods*.

### Phosphoproteomic Analysis.

Phosphopeptides were extracted from liver samples and analyzed on a liquid chromatography–mass spectrometry system. Detection, analysis, and validation protocols are described in *SI Appendix*, *Extended Materials and Methods*.

### Metabolomic Analysis.

Global metabolite profiling of liver, muscle, and plasma samples was performed by Metabolon. Metabolomic analysis is described in *SI Appendix*, *Extended Materials and Methods*.

## Supplementary Material

Supplementary File

Supplementary File

Supplementary File

## Data Availability

RNA-sequencing data that support the findings of this study have been deposited in Gene Expression Omnibus (accession code GSE176095). Metabolomics data are supplied in Dataset S2. Proteomics data have been deposited in the Proteomics Identification Database (identifier PXD032723).
