# Supplementary material for: Chronic inflammatory arthritis drives systemic changes in circadian energy metabolism
Source: Proc Natl Acad Sci U S A. 2022 Apr 28;119(18):e2112781119. doi: 10.1073/pnas.2112781119 (PMC9170023; doi:10.1073/pnas.2112781119)
Supplement: Supplementary File [file pnas.2112781119.sapp.pdf]

PNAS
www.pnas.org

Supplementary Information for

Chronic inflammatory arthritis drives systemic changes in circadian energy metabolism

Polly Downton[1], Fabio Sanna[2], Robert Maidstone[1,2], Toryn M. Poolman[2,5], Edward A. Hayter[1], Suzanna H. Dickson[1], Nick A. Ciccone[2], James O. Early[1], Antony Adamson[3], David G. Spiller[1], Devin A. Simpkins[1], Matthew Baxter[2], Roman Fischer[4], Magnus Rattray[1], Andrew S.I. Loudon[1], Julie E. Gibbs[1], David A. Bechtold[1]*, David W. Ray[2]*

[1] Centre for Biological Timing, Faculty of Biology, Medicine and Health, University of Manchester, Manchester, M13 9PT, UK

[2] NIHR Oxford Biomedical Research Centre, John Radcliffe Hospital, Oxford, UK and Oxford Centre for Diabetes, Endocrinology and Metabolism, University of Oxford, Oxford, OX3 9DU, UK

[3] Genome Editing Unit, Faculty of Biology, Medicine and Health, University of Manchester, Manchester, M13 9PT, UK

[4] Target Discovery Institute, Nuffield Department of Medicine, University of Oxford, OX3 7FZ, UK

[5] Present address: Structural & Molecular Biology, Faculty of Life Sciences, University College London, London, WC1E 6BT, UK

*Correspondence to: David A. Bechtold, david.bechtold@manchester.ac.uk; David W. Ray, david.ray@ocdem.ox.ac.uk

This PDF file includes:

Supplementary text
Figures S1 to S8
Tables S1 to S2
Legend for Datasets S1 to S3
Supplementary References

Other supplementary materials for this manuscript include the following:

Dataset S1
Dataset S2
Dataset S3

SUPPLEMENTARY TEXT

EXTENDED MATERIALS AND METHODS

Animals

Mice were maintained in the University of Manchester Biological Services Facility (BSF). DBA/1 mice were purchased from Envigo (Huntingdon, UK). Mice were group housed under a 12h:12h light/dark (L:D) cycle, at an ambient temperature of 22.5˚C +/-1˚C, and with food and water supplied *ad libitum*. All protocols were approved by the University of Manchester Animal Welfare and Ethical Review Body and licenced under the Animals (Scientific Procedures) Act 1986, UK Home Office project licence number P000BBBC3.

Collagen-induced arthritis (CIA)

Male DBA/1 mice (8-10 weeks) were administered bovine type II collagen emulsified in Complete Freund's Adjuvant (both MD Bioscience, Zurich, Switzerland) by intradermal injection under anaesthesia (2 x 50 μl of 1 mg/ml collagen in CFA). After 21 days, mice were given an intraperitoneal booster injection of collagen (200 μl of 1 mg/ml collagen in saline). Disease incidence and severity were tracked daily from day 18 post immunisation. Disease severity was scored on a four-point scale for each paw (1 for single inflamed digit, 2 for multiple inflamed digits, 3 for swelling of paw pad, 4 for severe swelling of paw pad and

joint) (1). Tissue collection occurred at ZT0, 4, 8, 12, 16 or 20 on day 7 after the development of disease symptoms in CIA mice, or at matched time points in naive control mice. Terminal blood was collected in MiniCollect K3EDTA tubes (Greiner Bio-One) and stored on ice prior to centrifugation at 3000 x *g* for 10 minutes at 4ºC. Plasma was transferred to a cryovial and flash frozen in liquid nitrogen. Tissue was transferred to cryovials and flash frozen in liquid nitrogen immediately after dissection.

Lipopolysaccharide (LPS) challenge

Female C57BL6 $Kmt2c^{flox}$ mice (aged 12-22 weeks) were housed in light controlled cages and exposed to 12h:12h L:D cycles for three weeks prior to the experiment. Mice were administered 1 mg/kg LPS (E.coli O127:B8, L4516, Sigma) in 0.9% sterile saline (6/time point), or saline only (3/time point), at ZT0 or ZT12 i.p. Tissue samples were collected four hours after challenge and flash frozen immediately.

In vivo phenotyping

Body composition was measured using an EchoMRI Body Composition Analyzer E26-258-MT machine (Echo Medical Systems). Activity and body temperature measurements were made following surgical implantation of radio telemetry devices (TA-F10, Data Sciences International) i.p. 7 to 10 days after the first CIA immunisation. Mice recovered for at least 4 days before being singly housed for telemetry recording. Measurements were recorded from day 14 after initial CIA immunisation for an asymptomatic period of up to one week, until booster immunisation on day 21. Recording was then resumed upon the development of symptoms. Food intake was monitored from day 14 by weighing remaining food pellets of singly housed mice at the start and end of each light phase.

Blood samples for measurement of fasting insulin and glucose measurement were collected eight days after the development of CIA symptoms. Food was withdrawn from the mice at ZT0. Blood was collected following removal of the tail tip at ZT8. Glucose concentration was measured immediately using an Aviva Accu-Chek meter (Roche). Remaining blood was centrifuged at 3000 x *g* for 10 minutes at

4ºC then stored at -80ºC for later analysis. Insulin level was measured by ELISA (Merck Millipore, EZRMI-13K) according to the manufacturer's instructions.

Histology

Paws for histological analysis were skinned, fixed overnight in formalin and then decalcified by incubation in Osteosoft Mild Decalcifier solution (VWR) for two weeks. Tissue was then paraffin embedded, sectioned and stained with either H&E (for cellular structures) or Safranin O (for cartilage) according to standard protocols. Slides were imaged by the University of Manchester Bioimaging Core Facility using a Pannoramic 250 Flash slide scanner (3DHistech) using a 20x/0.80 Plan Apochromat objective (Zeiss).

Cytokine analysis

For corticosterone measurements, serial tail blood samples were obtained over 48 hours. Blood was immediately centrifuged at 3000 x *g* for 10 minutes at 4ºC, then plasma was diluted 100-fold in PBS and frozen for later analysis using the Corticosterone ELISA kit (Abcam, ab108821). Terminal plasma samples were analysed using the BioPlex Pro Mouse Chemokine 33-plex panel (BioRad, catalogue reference 12002231). Plasma was diluted 1 in 4 in standard diluent buffer before mixing with assay beads. Samples were analysed on a BioPlex 200 machine. IL6 level was measured using the mouse IL6 ELISA kit (Abcam, ab100712). Plasma was diluted 1 in 4 in dilution buffer prior to application to the ELISA plate. Absorbance was measured using a GloMax Multi Detection System plate reader (Promega).

RNA analysis

RNA extraction

Joint tissue was ground with liquid nitrogen using a pestle and mortar, then transferred to a Lysing Matrix D tube containing Trizol. Tissue was homogenised

using a BeadMill homogeniser (3 x 4 m/s for 40s). RNA was extracted using chloroform then precipitated using isopropanol. After washing with 75% ethanol, the RNA pellet was resuspended in RNase-free water. RNA was purified from liver samples using the SV Total RNA kit (Promega), according to the manufacturer's instructions. Tissue was homogenised using Lysing Matrix D tubes loaded into a BeadMill homogeniser (4 m/s for 20s). RNA was eluted in RNase-free water. RNA was purified from muscle samples using the ReliaPrep Tissue kit (Promega), following the manufacturer's instructions for the purification of RNA from fibrous tissue. Muscle tissue was homogenised using the same protocol as for joint, and eluted in RNase-free water.

RNA-seq

Sequencing library preparation and sequencing was performed by the University of Manchester Genomic Technologies Core Facility. Sample quality was determined using a 2200 TapeStation (Agilent Technologies). Libraries were generated using the TruSeq Stranded mRNA assay (Illumina, Inc.) according to the manufacturer's protocol. The multiplexed libraries were analysed by paired-end sequencing on a HiSeq 4000 instrument (76 + 76 cycles, plus indices), then demultiplexed and converted using bcl2fastq software (v2.17.1.14, Illumina).

Data processing

Adaptors were removed and ends trimmed using Trimmomatic (v0.36), then reads were mapped against the mouse genome (mm10/GRCm38) using STAR (v2.5.3). Reads were counted, normalised and annotated in R using the Rsubread (v1.28.1), edgeR (v3.30.3) and biomaRt (v2.44.0) packages, respectively.

Data analysis

Differential expression analysis was run in R (2) using edgeR (v3.30.3). Genes were considered to the differentially expressed (DE) if the false discovery rate (FDR) was less than 1×10^{-20} (for joint) or less than 0.001 (for muscle and liver).

Differential rhythmicity analysis was performed using compareRhythms R package (v0.99.0, (3)). A model selection approach was used with genes being assigned to either arrhythmic, gain of rhythm, loss of rhythm, same rhythm in both, or a change in rhythm. A probability of being in a category of at least 0.6 was required for assignment. To avoid losing genes that were clearly differential rhythmic an extra category was used where the probability of either being a gain, loss or change in rhythm was greater than 0.6. Additional rhythmicity analysis was run using the JTK-cycle functionality of MetaCycle (v1.2.0), with period length fixed to 24 hours. Genes were considered to oscillate in naïve and/or CIA if the JTK-cycle adjP < 0.05 for one or both conditions.

For comparison of JTK-cycle and compareRhythms analysis of genes classified as losing or gaining rhythmicity with CIA, raw counts were normalised by subtracting the mean of each treatment (naïve or CIA), and dividing by the standard deviation across both treatments. Acrophase was calculated for each gene by fitting a sine wave (period constrained to 24 hours) to the normalised counts from the rhythmic group, and genes were aligned to this acrophase.
Pathway analysis of the gene lists defined above used the Enrichr web tool (4, 5) to detect significantly enriched pathways within the WikiPathways Mouse database (6). Raw analysis results are provided in Supplementary Dataset S3. Pathways are shown where adjusted p-value of enrichment < 0.05 and overlap with other significantly enriched pathways is under 30%. Fold enrichment was calculated relative to gene list size/total detected transcripts for the tissue of interest.

Upstream regulator analysis of the same gene lists was conducted using Ingenuity Pathway Analysis (Qiagen). Plots show the top twenty most significant upstream regulators belonging to the ‘Nuclear Receptor’ or ‘Transcriptional Regulator’ annotation classes. Percentage target detection is calculated relative to all direct ‘Expression’, ‘Transcription’ or ‘Protein-DNA’ interactions annotated in the Qiagen KnowledgeBase.

qRT-PCR

Liver RNA was converted to cDNA using the GoScript Reverse Transcriptase System kit (Promega A5001). Quantitative PCR was performed using KAPA SYBR fast universal mix (Kapa Biosystems KK4602) and a StepOnePlus Real-Time PCR machine (Applied Biosystems) with StepOne software v2.3. Primers (Sigma) are listed in Supplementary Table S2.

Phosphoproteomic analysis

Phosphoenrichment

Phosphoenrichment was done on an Agilent Bravo AssayMAP robot using Fe(III)-NTA cartridges (7) with slight adaptations. Cartridges were primed in ACN with 0.1% TFA and equilibrated with 80% ACN in 0.1% TFA. Peptides were loaded onto the cartridges followed by a wash with 80% ACN 0.1% TFA. Phosphopeptides were eluted with 1% NH3 and dried down in a vacuum centrifuge.

Mass spectrometry

Peptides were injected into a liquid chromatography-mass spectrometry (LC-MS) system comprised of a Dionex Ultimate 3000 nano LC and a Thermo Fusion Lumos. Peptides were separated on a 50-cm-long EasySpray column (ES803; Thermo Fisher) with a 75-µm inner diameter and a 60 minute gradient of 2 to 35% acetonitrile in 0.1% formic acid and 5% DMSO at a flow rate of 250 nL/min.

Data was acquired with the APD peak picking algorithm at a resolution of 120,000 and AGC target of 4e5 ions for a maximum injection time of 50 ms for MS1 spectra. The most abundant peaks were fragmented after isolation with a mass window of 1.6 Th with normalized collision energy 28% (HCD). MS2 spectra were acquired in the ion trap in rapid scan mode for a maximum injection time of 35 ms.

Phosphoproteome data analysis

The mass spectrometry proteomics data have been deposited to the ProteomeXchange Consortium via the PRIDE partner repository with the dataset identifier PXD032723 (8). RAW files were processed in Maxquant. Identified

phosphosites (phospho(STY).txt), were initially viewed and filtered in using the Perseus Framework. Potential contaminants and reverse peptides were removed. Phosphosites were filtered using a localisation probability of x>0.75, log2 transformed and further filtered to remove missing values, where sites with fewer than 15 valid (not N/A) values in a group (either CIA or naïve) were excluded. Missing values were imputed using random numbers drawn from a normal distribution with a width of 0.3 and down shift of 1.8. Ion intensities of identified phosphopeptides were normalized between each sample using trimmed means of M-values function from the edgeR (v3.30.3) R package. Differential phosphorylation analyses between groups were conducted using edgeR using a 5% FDR (9). Protein kinases analysis was performed using kinswingR package (10), using the curated kinase substrate sequences mouse dataset from PhosphoSitePlus (11). Phosphopeptide differential rhythmicity analysis was performed using compareRhythms (v0.99.0, (3)) in the same way as previously described for the RNA-seq data.

Western blotting

Mouse livers were lysed with western blot lysis buffer (NaCl 150mM (Sigma S3014-500G), Triton X-100 1% (Sigma T8787-50ML), SDS 0.1% (Sigma 74255-250G), Tris-HCl pH 8.0, 50 mM (Sigma T1503-500G), Sodium deoxycholate 0.5% (Sigma SRE0046-100G)), supplemented with protease and phosphatase inhibitors (Sigma 4906845001). Proteins were quantified with the Pierce BCA assay, and 10ug of proteins were boiled in NuPage loading buffer for 10 min and loaded into polyacrylamide Mini-PROTEAN TGX Gels (Biorad 4561096). Run performed at 100V constant voltage for 1h. Proteins were transferred onto nitrocellulose membrane using the Trans-Blot Turbo RTA Transfer Kit (Biorad 1704158). The membrane was blocked at room temperature for 2h with 5% milk in Tris-buffered saline, 0.1% tween 20 (TBS-T). The membranes were incubated with primary antibodies in TBS-T overnight at 4 °C, then with secondary antibody in TBS-T for 1h at RT. Primary antibodies used were: anti-pSTAT3 Tyr Y705 1:1000 (Cell Signaling 9145s), anti-pEGR Y1172 1:100 (MyBiosource MBS4152963), anti-

STAT3 1:1000 (Cell Signaling 9139T), anti-EGFR 1:1000 (Life Technologies PA11110) and anti-GAPDH−Peroxidase 1:30000 (Sigma G9295-200UL). Secondary antibodies were: CF660C goat anti-Mouse IgG (H+L) 1:10000 (Insight Biotechnology 20050-1), goat anti-Rabbit IgG (H+L) HRP conjugated 1:1000 (Thermo Fisher 32460). HRP conjugated secondary antibody binding was detected with the Radiance Plus chemiluminescence kit (Cambridge Bioscience AC2103).

Metabolomic analysis

Global metabolite profiling of liver, muscle and plasma samples was performed by Metabolon (Durham, NC, USA). Samples were analysed using the HD4 platform, which uses ultrahigh performance liquid chromatography- tandem mass spectrometry (UPLC-MS/MS) methods for metabolite detection. Peaks were quantified by area under the curve, and normalised to set metabolite median value to one. Analysis by two-way ANOVA was used to identify metabolites showing altered level with treatment and/or time. Metabolites were considered to be significantly altered at a time point if the one-way ANOVA contrast false discovery rate (q-value) was < 0.05. Metabolomic summary data and details of statistical tests are provided in Supplementary Dataset S2. Metabolite differential rhythmicity analysis was performed using CompareRhythms (v0.99.0,(3)) in the same way as previously described for the RNA-seq data.

For inter and intra tissue metabolite correlation plots, Pearson's linear correlation was calculated for data averaged at each time point for every pair of metabolites. Using a stringent threshold ($P<0.001$), every metabolite in a given family that correlated with at least one metabolite in a target family contributed to ribbon width between the two families. Segment size reflects the number of detected metabolites in each tissue. Ribbons with width <10% of family size at both ends were omitted for clarity. Data was processed in MATLAB R2019a (Mathworks, US) and plots made using the Circlize visualisation package in R (12).

Statistics

Statistical tests and sample numbers are specified in figure legends where appropriate. Full details of statistical tests are provided in Supplementary Dataset S1. Statistical tests were conducted in GraphPad Prism. Throughout, * denotes p<0.05, ** denotes p<0.01, *** denotes p<0.001 and **** denotes p<0.0001. Plots were produced in GraphPad Prism, using the R package ggplot2 (13), or using Matlab.

SUPPLEMENTAL DISCUSSION AND STUDY LIMITATIONS

Characterising rhythmic processes is complex with numerous methods available, each with strengths and caveats. Here we use a comparative analytical approach (CompareRhythms, (3)), which has improved implementation of cosinor regression for comparing rhythmicity directly between conditions. Utilising cosinor analysis and model selection, as done within CompareRhythms, is a much needed development in differential rhythmicity analysis. JTK-cycle (14) and similar methods such as RAIN (15) are still commonly used for this kind of comparison. However, these methods are not built for identifying differences in rhythmicity between conditions. JTK-cycle, RAIN and similar methods are built for rhythm detection based on samples from one condition. Using them for differential rhythm detection by comparing 'rhythmic' gene lists from each condition (analysed separately) leads to a high rate of false discovery (16); for example, just achieving significance in one sample, and just failing to reach significance in another, results in the incorrect inference that they are different from each other.

Model selection methods such as CompareRhythms (3) and dryR (17) classify paired gene profiles into distinct categories: arrhythmic, loss of rhythm, gain of rhythm, same rhythm or changed rhythm. The gene profiles are then modelled jointly across the two conditions and probability scores for membership of each category obtained. Whilst we have predominantly used this comparative analysis approach, we have also included analyses with more traditional methods (JTK

cycle) to make our study fully comparable with previous work. Both methods estimate similar numbers of genes to be rhythmic overall, and identify similar changes in rhythmicity, functional pathway enrichment and potential upstream regulators. This is robust across different expression and probability thresholds. Nevertheless, we must acknowledge that using different thresholds and/or analysis approaches will change the number of rhythmic genes detected within a tissue and given condition. Indeed, drawing conclusions based on absolute gene numbers assigned to any given category (rhythmic, gain, loss etc) should be avoided. Importantly, our gene ontology analyses and upstream regulator analyses were consistent across multiple methods, expression level cut-offs and probability thresholds.

In this study, we analyse rhythmic changes in a complex disease model which can show considerable differences between individual mice. Therefore, variability in disease state between mice could influence the assessment of differential expression and rhythmicity between naïve and CIA mice. To mitigate this, we implemented strict criteria regarding disease severity for sample inclusion (Figure 1A), and include robust numbers of replicate samples collected over multiple independent experimental runs for each time point. Our transcriptomic and metabolomic samples were collected from four independent experimental mouse cohorts, and standardised to ensure disease severity was evenly distributed across time points and replicates. We characterised five samples at each time point to minimise the risk of false positive detection due to noise and biological variability (18).

We cannot rule out that behavioural effects associated with CIA (such as reduced locomotor activity) may have contributed to transcriptional and/or metabolic differences between naïve and CIA mice. Our telemetric assessment of activity and body temperature suggests that overall levels of activity are reduced in CIA mice once they develop symptomatic disease; however, there remains a significant difference in activity between the dark and light phases. Due to the method of

activity measure (radio telemetry using DSI TA-F10 remotes), we cannot determine absolute activity levels in the animals (as the activity counts generated are not directly proportional to distance travelled). Importantly, we show that physiological measures (body temperature) and peripheral entraining signals (corticosterone) remain robustly rhythmic in these animals even during symptomatic disease.

Previous studies have found that exercise can entrain the mouse circadian clock (15), and can alter rhythmic physiology and clock gene expression in peripheral tissues (16), including skeletal muscle (17). In light of these findings, it is notable that we do not observe changes in rhythmicity of the core clock genes in muscle tissue with disease (Supplementary Figure S3C). We therefore consider it to be much more likely that the changes we observe in clock gene expression in the joint, the primary site of inflammation, are attributable to disease processes rather than any loss of activity-related entrainment.

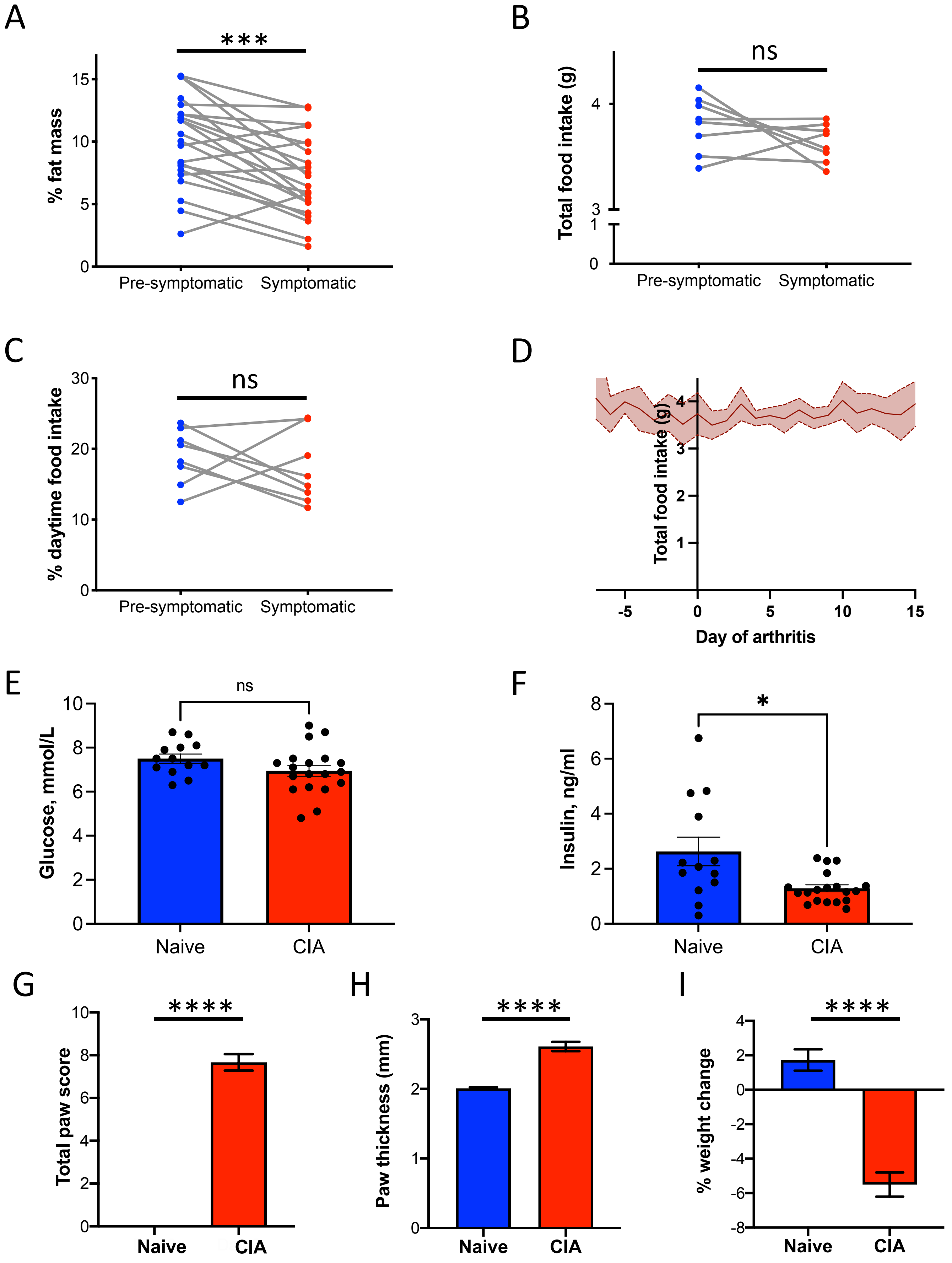

Supplementary Figure 1.

Supplementary Figure 1

A. Body composition was assessed by EchoMRI measurement of % fat mass before and after the emergence of arthritic symptoms. Presymptomatic measurements were made between days -10 and -3, symptomatic measurements were made between days 2 and 7 (paired Wilcoxon test, n = 22). **B,C**. Average daily food intake and % light phase food intake were measured over presymptomatic days -3 to -1 and symptomatic days 5 to 7 (paired two-tailed t-test, n = 8). **D**. Total food intake measurements over the course of arthritic symptom development (n = 9). **E,F.** Fasting blood glucose and insulin measurements from naïve and CIA mice. Welch's unpaired t-test, n = 13 (naïve) and 19 (CIA). Error bars represent mean +/- SEM. **G,H,I**. Day 6 (day prior to collection) paw score, paw thickness and weight measurements for experimental samples selected for tissue analysis (unpaired t test or Wilcoxon test, n = 28-30 per condition).

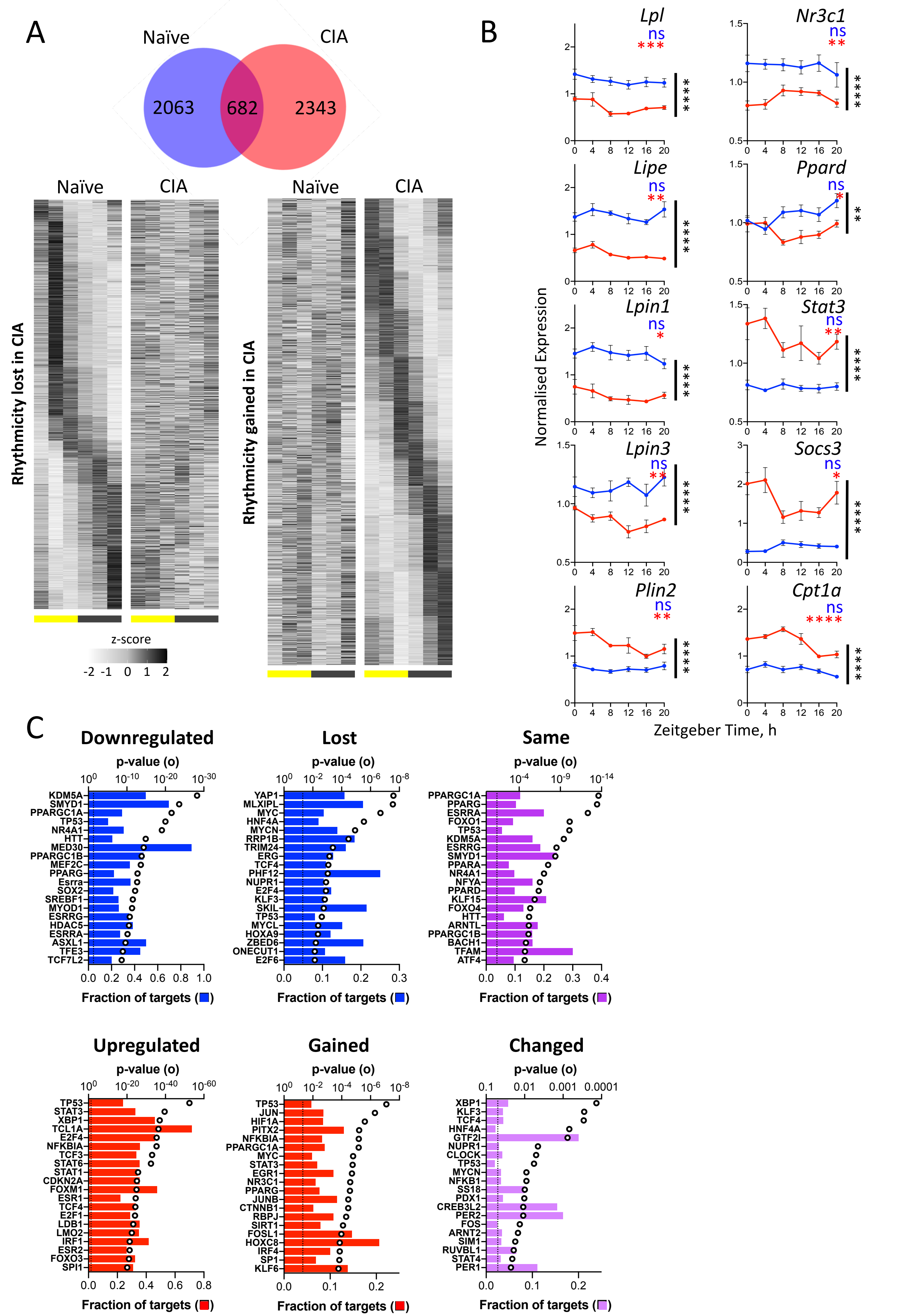

Supplementary Figure 2.

Supplementary Figure 2

A. Differential rhythmicity analysis using JTK-cycle was used to group joint transcript expression profiles according to change or maintenance of rhythmicity with disease. Venn diagram shows the intersect of these groups. Heatmaps represent the normalised (z-scored) transcript expression levels in naïve (left) and CIA (right) mice over time (columns, from ZT0 at 4h intervals). **B**. Normalised transcript expression profiles of genes involved in fatty acid metabolism and signalling (treatment effect on two-way ANOVA indicated to right of each profile; JTK-cycle adjusted p-value for rhythmicity indicated in blue/red above each profile). **C**. Upstream regulator analysis of joint RNAseq data using Ingenuity IPA. Top 20 most significant upstream regulators are shown for each category (open circle represents significance of enrichment, filled bar represents fraction of downstream targets of the regulator found in the category, dashed line indicates P = 0.05).

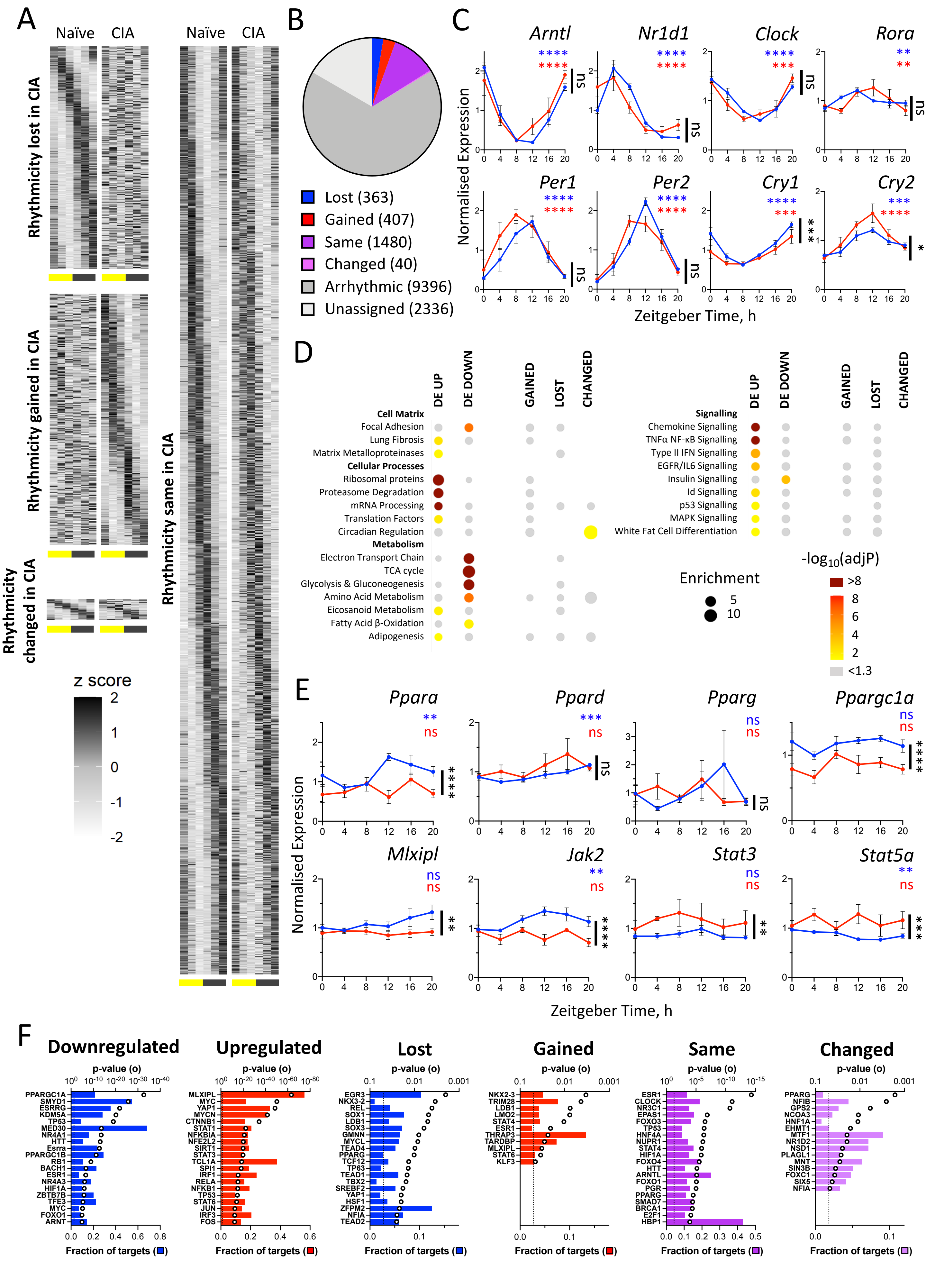

Supplementary Figure 3.

Supplementary Figure 3

A, B. Matched muscle samples were analysed by RNAseq to characterize the effect of distal inflammatory disease. Differential rhythmicity analysis with compareRhythms categorised transcript expression profiles according to change or maintenance of rhythmicity with disease. Heatmaps represent the normalised (z-scored) transcript expression levels in naïve (left) and CIA (right) mice over time (columns, from ZT0 at 4h intervals). **C**. Rhythmic expression of most core clock genes was maintained in muscle from CIA mice (CIA versus naïve comparison by two-way ANOVA in black; adjusted p-value of JTK analysis in blue/red; n=5/point, *P<0.05, **P<0.01, ***P<0.001, ****P<0.0001). **D**. Groups of genes showing significant differential expression (DE) or rhythmic change with disease were analysed for functional enrichment using the Enrichr tool (see Methods for details). Spot size represents fold enrichment of genes in group versus the genome; colour represents significance of enrichment; spot absence means no genes from the pathway were allocated to the group on statistical categorisation. **E**. Transcript expression profiles of genes involved in the EGFR and nuclear receptor signalling pathways showed loss of rhythmicity and/or differential expression with CIA. Data is presented as mean +/- SEM throughout. **F**. Upstream regulator analysis of muscle transcripts showing altered expression with CIA using Ingenuity IPA. Top 20 most significant upstream regulators, or all upstream regulators with P < 0.05, are shown for each category (open circle represents significance of enrichment, filled bar represents fraction of downstream targets of the regulator found in the category, dashed line indicates P = 0.05).

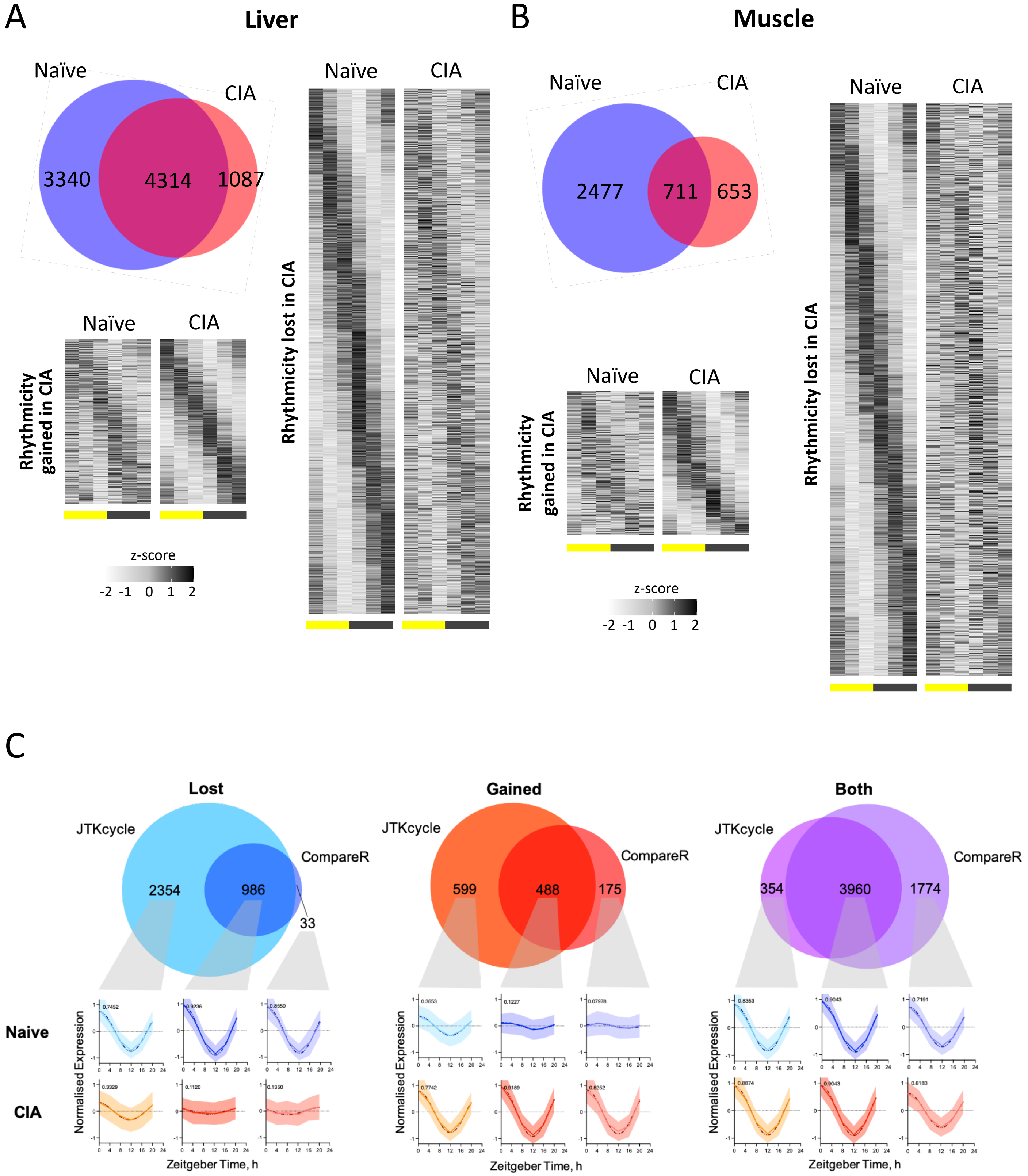

Supplementary Figure 4.

Supplementary Figure 4

A. Rhythmicity analysis with JTK-cycle was used to group liver transcript expression profiles according to change or maintenance of rhythmicity with disease. Venn diagram shows the intersect of these groups. Heatmaps represent the normalised (z-scored) transcript expression levels in naïve (left) and CIA (right) mice over time (columns, from ZT0 at 4h intervals). **B**. Rhythmicity analysis with JTK-cycle was used to group muscle transcript expression profiles according to change or maintenance of rhythmicity with disease. Data is presented as in A. **C**. Comparison of rhythmic gene identification by JTK-cycle and compareRhythms. Venn diagrams (top) compare the number of genes characterised as having lost (left) or gained (centre) rhythmicity with CIA, or which were rhythmic in both conditions (right) as determined by JTK-cycle and compareRhythms analysis. Profiles (below) represent average normalised expression of genes within each Venn diagram segment, aligned to the predicted acrophase of each gene (solid line, shading represents standard deviation). Fitting by nonlinear regression (dashed line) was used to calculate amplitude of the average profile, indicated on the top left of each plot.

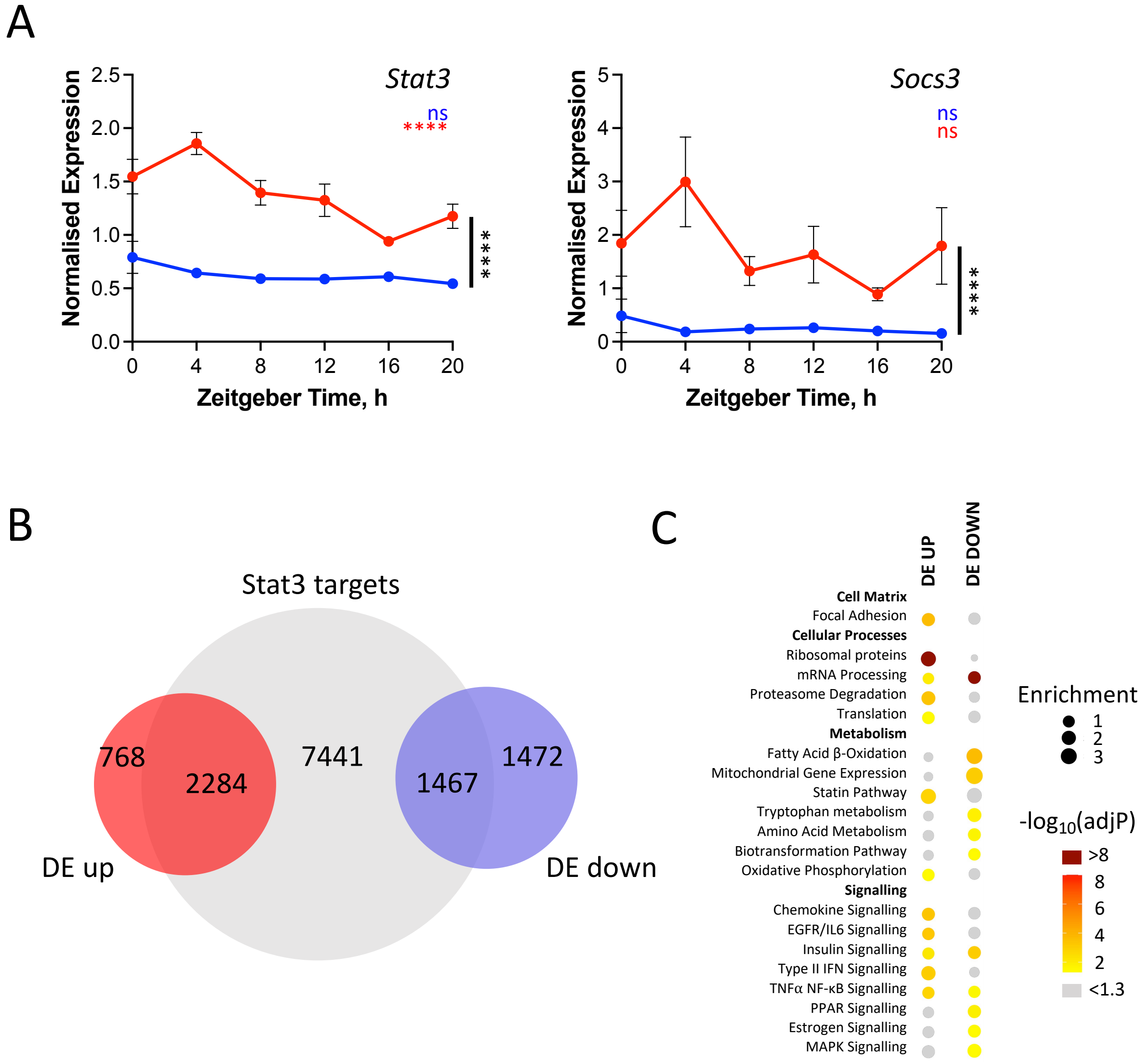

Supplementary Figure 5.

Supplementary Figure 5

A. Normalised transcript expression profiles of genes involved in EGFR-responsive signalling in naïve (blue) and CIA (red) liver. Treatment effect on two-way ANOVA is indicated to right of each profile, JTK-cycle adjusted p-value is indicated to the top right of each profile. Data is presented as mean +/- SEM. **B**. Venn diagram demonstrating the overlap between genes differentially expressed in CIA mice and targets of STAT3 binding, as determined by chromatin immunoprecipitation (19). Cistrome data was extracted from CistromeDB (20). STAT3 targets were defined as genes for which the cistromeDB score was greater than zero in at least one IL6-treated hepatocyte sample. **C**. Enrichr analysis of STAT3 target genes that are differentially regulated in CIA mice compared to naïve controls. Spot size represents fold enrichment of genes in group versus the genome; colour represents significance of enrichment; spot absence means no genes from the pathway were allocated to the group on statistical categorisation.

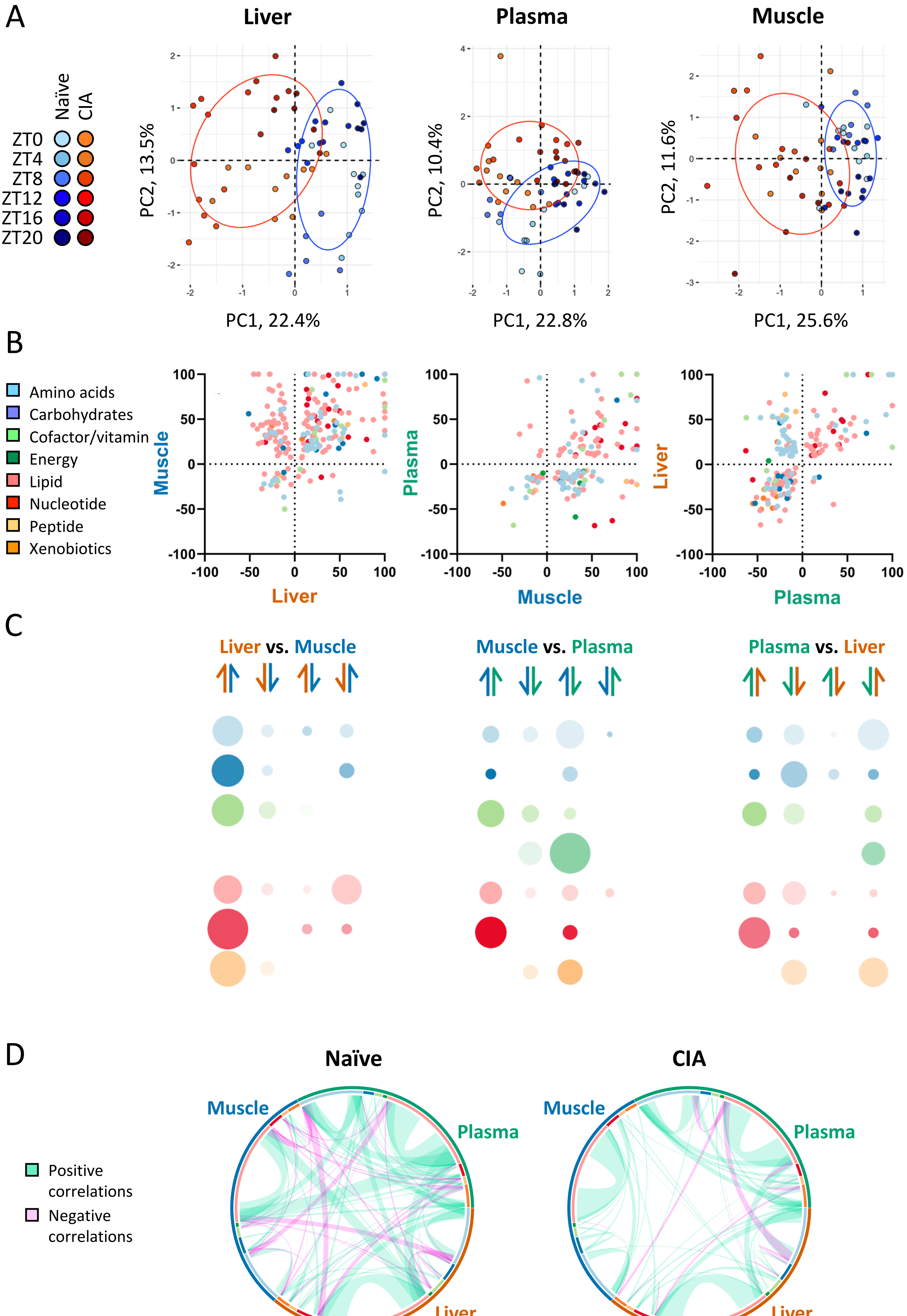

Supplementary Figure 6.

Supplementary Figure 6

A. Principle component analysis of metabolite profiles in liver, plasma and muscle. **B**. Quadrant plot showing correlation of metabolite changes between different tissues. Axes represent % change in metabolite level, where positive values represent metabolites upregulated in CIA. Only significantly altered metabolites are shown. Colour represents metabolite family. Axes are curtailed at ±100% and metabolites that lie outside of these limits are shown at 100%. **C.** Bubble plots representing metabolite families in each quadrant in B. Arrows dictate the quadrant and whether metabolites are up or down regulated in each tissue (colour). Circle size represents the proportion of the metabolite family in each quadrant and depth of colour represents average magnitude of change between tissues. **D**. Inter- and intra-tissue temporal correlation between metabolite families in naïve (left), and arthritic (right) mice). Circle edge represents tissue (outer ring) and metabolite family (inner ring). Connecting ribbon thickness indicates proportion of metabolites within the family that significantly correlate with at least one member of the connecting family ($p<0.001$). Ribbons of width <10% of family size on both ends were omitted for clarity.

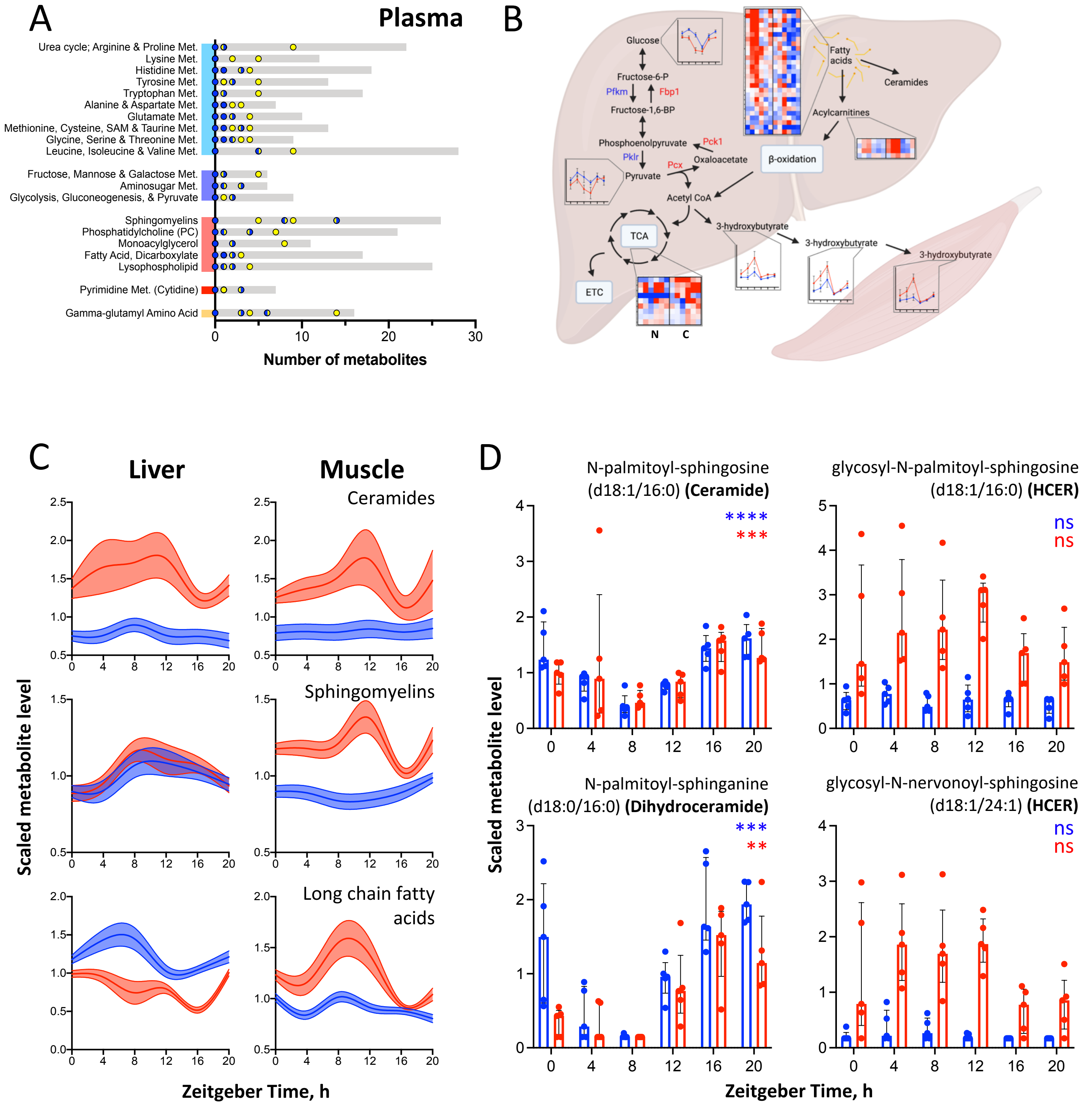

Supplementary Figure 7.

Supplementary Figure 7

A. Metabolite subpathways sorted by average differential metabolite detection between light (ZT4, 8, 12) and dark (ZT16, 20, ZT0) conditions in plasma (grey bars represent total detected metabolites, points represent number of differentially detected metabolites at light (yellow), dark (blue) or transition (yellow and blue) time points). **B**. Schematic representation of energy metabolism and ketone synthesis pathways integrating differential gene expression (named genes; red/blue indicate significant up/downregulation respectively) and metabolite detection profiles (normalised level +/- SEM represent single metabolites; heatmaps represent change in groups of selected metabolites in naïve (left, N) and CIA (right, C) mice over time from ZT0 to ZT20; n = 5 per condition). Created with BioRender.com. **C**. Spline graphs indicating the detection profiles of scaled metabolite level for selected significantly altered subpathways involved in lipid metabolism; error bars give 95% confidence interval around the mean. The 'ceramide' group includes all metabolites belonging to the dihydroceramide, ceramide and hexosylceramide subpathways; the 'sphingomyelin' group includes all metabolites from the dihydrosphingomyelin and sphingomyelin subpathways; the 'long chain fatty acid' group includes all saturated, monounsaturated and polyunsaturated fatty acids. **D**. Normalised levels of ceramide-related metabolites detected in plasma (median with interquartile range, n = 5 per condition, adjusted p-value of JTK analysis indicated in blue/red).

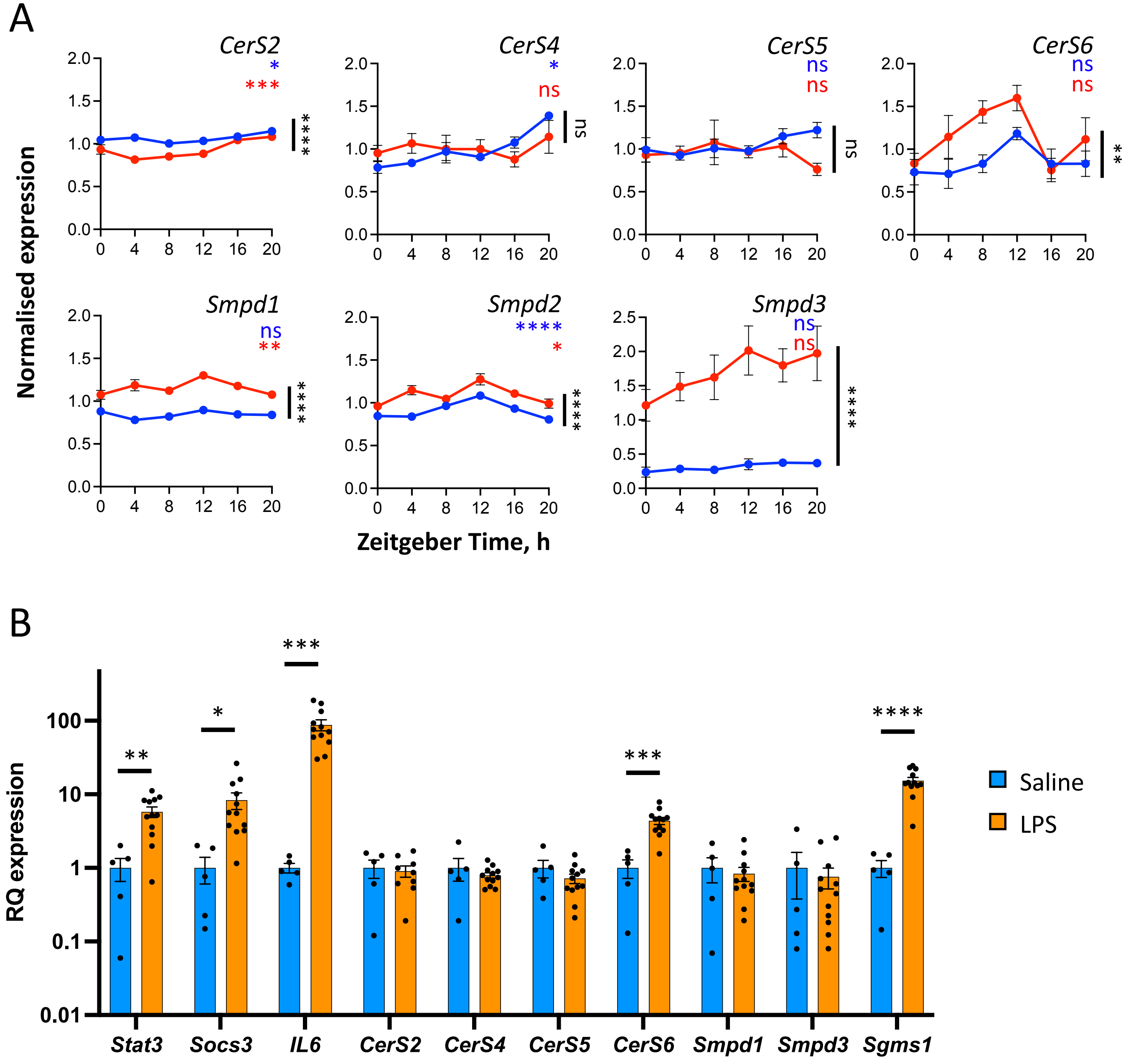

Supplementary Figure 8.

Supplementary Figure 8

A. Transcript levels for lipid metabolism genes in liver of naïve (blue) and CIA (red) mice. Values are normalized to the average expression level of the gene across all conditions. Error bars represent SEM, n = 5 per condition. **B**. qRT-PCR analysis of gene expression changes in liver from mice exposed to acute i.p. LPS treatment (orange, n = 12), or saline control treatment (turquoise, n = 5). Relatively quantified (RQ) expression is normalised to *Ppia* and shown as fold change compared to average saline treatment. Error bars represent SEM.

Table S1. Candidate kinases mediating inflammatory disease response of the liver phosphoproteome. Table of kinases predicted to be involved in differential phosphorylation of the liver proteome in response to CIA, as identified by the KinSwingR tool (see Extended Methods) and sorted by Normalised KinSwing score, which integrates observed changes in phosphorylation to the substrates of the kinase, correcting for the number of targets containing the target substrate motif (position weight matrix, PWM). Kinases directly identified in our dataset (29/49) are highlighted in grey. Kinases that showed differential phosphorylation between naïve and CIA conditions (7/49) are highlighted in red text.

Kinase	Number of Substrates			Fraction of Substrates		Raw KinSwing Score	Substrates in kinase PWM	Normalised KinSwing Score	Probability of greater score	Probability of lesser score
	Positive	Negative	All	Positive	Negative					
RSK2	59	38	97	0.608	0.392	15.949	14	2.118	0.010	0.980
Nik	*52*	*36*	*88*	*0.591*	*0.409*	*13.047*	*14*	*1.757*	*0.020*	*0.970*
Abl	59	44	103	0.573	0.427	12.801	23	1.727	0.040	0.960
PRKD1	*56*	*40*	*96*	*0.583*	*0.417*	*10.619*	*10*	*1.455*	*0.040*	*0.960*
**PKCA**	**57**	**49**	**106**	**0.538**	**0.462**	**9.086**	**73**	**1.265**	**0.129**	**0.861**
**Akt1**	**52**	**44**	**96**	**0.542**	**0.458**	**8.558**	**42**	**1.199**	**0.158**	**0.842**
Src	*60*	*53*	*113*	*0.531*	*0.469*	*7.694*	*79*	*1.092*	*0.208*	*0.782*
PKCB	56	45	101	0.554	0.446	7.267	11	1.039	0.050	0.941
CAMK2A	*49*	*44*	*93*	*0.527*	*0.473*	*5.923*	*57*	*0.871*	*0.188*	*0.802*
**AMPKA1**	**48**	**43**	**91**	**0.527**	**0.473**	**5.068**	**30**	**0.765**	**0.218**	**0.792**
ERK2	*49*	*46*	*95*	*0.516*	*0.484*	*4.547*	*193*	*0.700*	*0.257*	*0.713*
PKCZ	49	43	92	0.533	0.467	4.407	12	0.683	0.149	0.832
CDK2	*47*	*44*	*91*	*0.516*	*0.484*	*3.250*	*38*	*0.539*	*0.297*	*0.703*
PKCD	52	48	100	0.520	0.480	2.997	15	0.508	0.287	0.713
SGK1	*49*	*45*	*94*	*0.521*	*0.479*	*2.786*	*11*	*0.481*	*0.238*	*0.733*
ATM	*44*	*41*	*85*	*0.518*	*0.482*	*2.612*	*16*	*0.460*	*0.317*	*0.644*
CK2A1	*46*	*44*	*90*	*0.511*	*0.489*	*2.396*	*54*	*0.433*	*0.337*	*0.663*
PKCT	50	47	97	0.515	0.485	2.038	11	0.388	0.356	0.644
CDK5	*43*	*42*	*85*	*0.506*	*0.494*	*1.376*	*80*	*0.306*	*0.396*	*0.604*
p90RSK	55	53	108	0.509	0.491	1.374	14	0.306	0.396	0.604
LYN	53	52	105	0.505	0.495	0.877	27	0.244	0.386	0.624
MAPKAPK2	*47*	*46*	*93*	*0.505*	*0.495*	*0.812*	*16*	*0.236*	*0.475*	*0.515*
CDK4	47	46	93	0.505	0.495	0.702	11	0.222	0.376	0.554
AurB	*48*	*48*	*96*	*0.500*	*0.500*	*0.000*	*14*	*0.135*	*0.426*	*0.495*
JNK2	39	39	78	0.500	0.500	0.000	15	0.135	0.465	0.455
PKG1	43	43	86	0.500	0.500	0.000	16	0.135	0.495	0.426
PLK4	*44*	*45*	*89*	*0.494*	*0.506*	*-0.777*	*13*	*0.038*	*0.505*	*0.505*
**EGFR**	**43**	**44**	**87**	**0.494**	**0.506**	**-0.855**	**16**	**0.029**	**0.455**	**0.515**
PKCE	46	48	94	0.489	0.511	-1.645	17	-0.070	0.624	0.376
CDK1	*43*	*45*	*88*	*0.489*	*0.511*	*-2.285*	*42*	*-0.149*	*0.624*	*0.366*
ROCK2	*43*	*47*	*90*	*0.478*	*0.522*	*-2.767*	*10*	*-0.209*	*0.594*	*0.406*
Lck	*46*	*50*	*96*	*0.479*	*0.521*	*-3.479*	*21*	*-0.298*	*0.634*	*0.356*
**p70S6K**	**45**	**50**	**95**	**0.474**	**0.526**	**-4.082**	**17**	**-0.373**	**0.634**	**0.356**
**Met**	**47**	**53**	**100**	**0.470**	**0.530**	**-4.128**	**12**	**-0.378**	**0.683**	**0.297**
MSK1	41	48	89	0.461	0.539	-5.095	11	-0.499	0.733	0.267
PDK1	45	53	98	0.459	0.541	-5.598	12	-0.561	0.812	0.168
JNK3	*45*	*54*	*99*	*0.455*	*0.545*	*-6.251*	*12*	*-0.642*	*0.832*	*0.149*
CK1D	44	50	94	0.468	0.532	-6.715	47	-0.700	0.644	0.366
IKKB	44	53	97	0.454	0.546	-7.389	18	-0.784	0.871	0.129
P38A	44	51	95	0.463	0.537	-8.232	59	-0.889	0.634	0.347
JNK1	*45*	*54*	*99*	*0.455*	*0.545*	*-9.280*	*40*	*-1.019*	*0.802*	*0.188*
GSK3B	38	46	84	0.452	0.548	-10.450	61	-1.165	0.871	0.119
Syk	*38*	*51*	*89*	*0.427*	*0.573*	*-10.466*	*14*	*-1.167*	*0.881*	*0.119*
Fyn	37	47	84	0.440	0.560	-11.224	34	-1.261	0.901	0.089
PLK1	*39*	*58*	*97*	*0.402*	*0.598*	*-12.553*	*10*	*-1.426*	*0.970*	*0.010*
**mTOR**	**41**	**51**	**92**	**0.446**	**0.554**	**-13.166**	**85**	**-1.502**	**0.871**	**0.099**
PKACA	41	51	92	0.446	0.554	-15.113	164	-1.745	0.812	0.129
JAK2	41	59	100	0.410	0.590	-16.588	27	-1.928	0.941	0.030
ERK1	36	52	88	0.409	0.591	-21.211	73	-2.503	0.931	0.050

Table S2. Reagents for qRT-PCR.

Table of primer pairs and probes used for qRT-PCR analysis of mouse liver samples.

Target	Forward Primer (5’ to 3’)	Reverse Primer (5’ to 3’)
Ppia	TATCTGCACTGCCAAGACTGAGTG	CTTCTTGCTGGTCTTGCCATTCC
Stat3	AGAACCTCCAGGACGACTTTG	TCACAATGCTTCTCCGCATCT
Socs3	GCACAAGCACAAAAATCCAGC	AGAAGCCAATCTGCCCCTG
IL6	CCGGAGAGGAGACTTCACAGA	AGAATTGCCATTGCACAACTCTT
Cers2	GAAGTGGGAAACGGAGTAGCG	AGTTCACAGGCAGCCATAGTCG
Cers4	CTGTGGTACTGTTGTTGCATGAC	GCGCGTGTAGAAGAAGACTAAG
Cers5	TGGCCAATTATGCCAGACGTGAG	GGTAGGGCCCAATAATCTCCCAGC
Cers6	GCATTCAACGCTGGTTTCGAC	TTCAAGAACCGGACTCCGTAG
Smpd1	TGGGACTCCTTTGGATGGG	CGGCGCTATGGCACTGAAT
Smpd3	ACACGACCCCCTTTCCTAATA	GGCGCTTCTCATAGGTGGTG
Sgms1	CATTTCAACTGTTCTCCGAAGC	CCATCGTGTGATACCACCAG

LEGEND FOR SUPPLEMENTARY DATASETS

Dataset S1. Summary of statistical analyses.

This dataset provides details of statistical tests used to analyse data presented in Figures and Supplementary Figures in this manuscript.

Dataset S2. Summary of metabolomic analyses.

This dataset provides summary data of metabolomic analysis of liver, plasma and muscle samples.

Dataset S3. Summary of gene enrichment analyses.

This dataset provides the raw results obtained by Enrichr analysis of gene lists discussed in this manuscript. Gene lists were queried against the Wikimouse Pathways 2019 database.

References

1. L. E. Hand *et al.*, The circadian clock regulates inflammatory arthritis. *FASEB J* **30**, 3759-3770 (2016).
2. R Core Team, *R: A language and environment for statistical computing* (R Foundation for Statistical Computing, Vienna, Austria., 2020).
3. A. Pelikan, H. Herzel, A. Kramer, B. Ananthasubramaniam, Venn diagram analysis overestimates the extent of circadian rhythm reprogramming. *FEBS J* 10.1111/febs.16095 (2021).
4. E. Y. Chen *et al.*, Enrichr: interactive and collaborative HTML5 gene list enrichment analysis tool. *BMC Bioinformatics* **14**, 128 (2013).
5. M. V. Kuleshov *et al.*, Enrichr: a comprehensive gene set enrichment analysis web server 2016 update. *Nucleic Acids Res* **44**, W90-97 (2016).
6. D. N. Slenter *et al.*, WikiPathways: a multifaceted pathway database bridging metabolomics to other omics research. *Nucleic Acids Res* **46**, D661-D667 (2018).
7. H. Post *et al.*, Robust, Sensitive, and Automated Phosphopeptide Enrichment Optimized for Low Sample Amounts Applied to Primary Hippocampal Neurons. *J Proteome Res* **16**, 728-737 (2017).
8. Y. Perez-Riverol *et al.*, The PRIDE database resources in 2022: a hub for mass spectrometry-based proteomics evidences. *Nucleic Acids Res* **50**, D543-D552 (2022).
9. L. D. Wang *et al.*, Phosphoproteomic profiling of mouse primary HSPCs reveals new regulators of HSPC mobilization. *Blood* **128**, 1465-1474 (2016).
10. K. Engholm-Keller *et al.*, The temporal profile of activity-dependent presynaptic phospho-signalling reveals long-lasting patterns of poststimulus regulation. *PLoS Biol* **17**, e3000170 (2019).
11. P. V. Hornbeck *et al.*, PhosphoSitePlus, 2014: mutations, PTMs and recalibrations. *Nucleic Acids Res* **43**, D512-520 (2015).
12. Z. Gu, L. Gu, R. Eils, M. Schlesner, B. Brors, circlize Implements and enhances circular visualization in R. *Bioinformatics* **30**, 2811-2812 (2014).
13. H. Wickham, *ggplot2: Elegant Graphics for Data Analysis.* (Springer-Verlag New York, 2016).
14. M. E. Hughes, J. B. Hogenesch, K. Kornacker, JTK_CYCLE: an efficient nonparametric algorithm for detecting rhythmic components in genome-scale data sets. *J Biol Rhythms* **25**, 372-380 (2010).
15. P. F. Thaben, P. O. Westermark, Detecting rhythms in time series with RAIN. *J Biol Rhythms* **29**, 391-400 (2014).
16. P. F. Thaben, P. O. Westermark, Differential rhythmicity: detecting altered rhythmicity in biological data. *Bioinformatics* **32**, 2800-2808 (2016).
17. B. D. Weger *et al.*, Systematic analysis of differential rhythmic liver gene expression mediated by the circadian clock and feeding rhythms. *Proc Natl Acad Sci U S A* **118** (2021).

18. S. Y. Krishnaiah *et al.*, Clock Regulation of Metabolites Reveals Coupling between Transcription and Metabolism. *Cell Metab* **25**, 961-974 e964 (2017).
19. I. Goldstein, V. Paakinaho, S. Baek, M. H. Sung, G. L. Hager, Synergistic gene expression during the acute phase response is characterized by transcription factor assisted loading. *Nat Commun* **8**, 1849 (2017).
20. R. Zheng *et al.*, Cistrome Data Browser: expanded datasets and new tools for gene regulatory analysis. *Nucleic Acids Res* **47**, D729-D735 (2019).
